# Induced Immune Reaction in the Acorn Worm, *Saccoglossus kowalevskii*, Informs the Evolution of Antiviral Immunity

**DOI:** 10.1093/molbev/msad097

**Published:** 2023-04-28

**Authors:** Michael G Tassia, Haley A Hallowell, Damien S Waits, Ryan C Range, Christopher J Lowe, Rita M Graze, Elizabeth Hiltbold Schwartz, Kenneth M Halanych

**Affiliations:** Department of Biological Sciences, Auburn University, Auburn, AL; Department of Biology, Johns Hopkins University, Baltimore, MD; Department of Biological Sciences, Auburn University, Auburn, AL; W. Harry Feinstone Department of Molecular Microbiology and Immunology, Johns Hopkins Bloomberg School of Public Health, Baltimore, MD; Department of Biological Sciences, Auburn University, Auburn, AL; Center for Marine Science, University of North Carolina Wilmington, Wlimington, NC; Department of Biological Sciences, Auburn University, Auburn, AL; Hopkins Marine Station, Stanford University, Pacific Grove, CA; Department of Biological Sciences, Auburn University, Auburn, AL; Department of Biological Sciences, Auburn University, Auburn, AL; Department of Biological Sciences, Auburn University, Auburn, AL; Center for Marine Science, University of North Carolina Wilmington, Wlimington, NC

**Keywords:** deuterostome, immunity, differential expression, evolution

## Abstract

Evolutionary perspectives on the deployment of immune factors following infection have been shaped by studies on a limited number of biomedical model systems with a heavy emphasis on vertebrate species. Although their contributions to contemporary immunology cannot be understated, a broader phylogenetic perspective is needed to understand the evolution of immune systems across Metazoa. In our study, we leverage differential gene expression analyses to identify genes implicated in the antiviral immune response of the acorn worm hemichordate, *Saccoglossus kowalevskii*, and place them in the context of immunity evolution within deuterostomes—the animal clade composed of chordates, hemichordates, and echinoderms. Following acute exposure to the synthetic viral double-stranded RNA analog, poly(I:C), we show that *S. kowalevskii* responds by regulating the transcription of genes associated with canonical innate immunity signaling pathways (e.g., nuclear factor κB and interferon regulatory factor signaling) and metabolic processes (e.g., lipid metabolism), as well as many genes without clear evidence of orthology with those of model species. Aggregated across all experimental time point contrasts, we identify 423 genes that are differentially expressed in response to poly(I:C). We also identify 147 genes with altered temporal patterns of expression in response to immune challenge. By characterizing the molecular toolkit involved in hemichordate antiviral immunity, our findings provide vital evolutionary context for understanding the origins of immune systems within Deuterostomia.

## Introduction

**Fig. 1.**
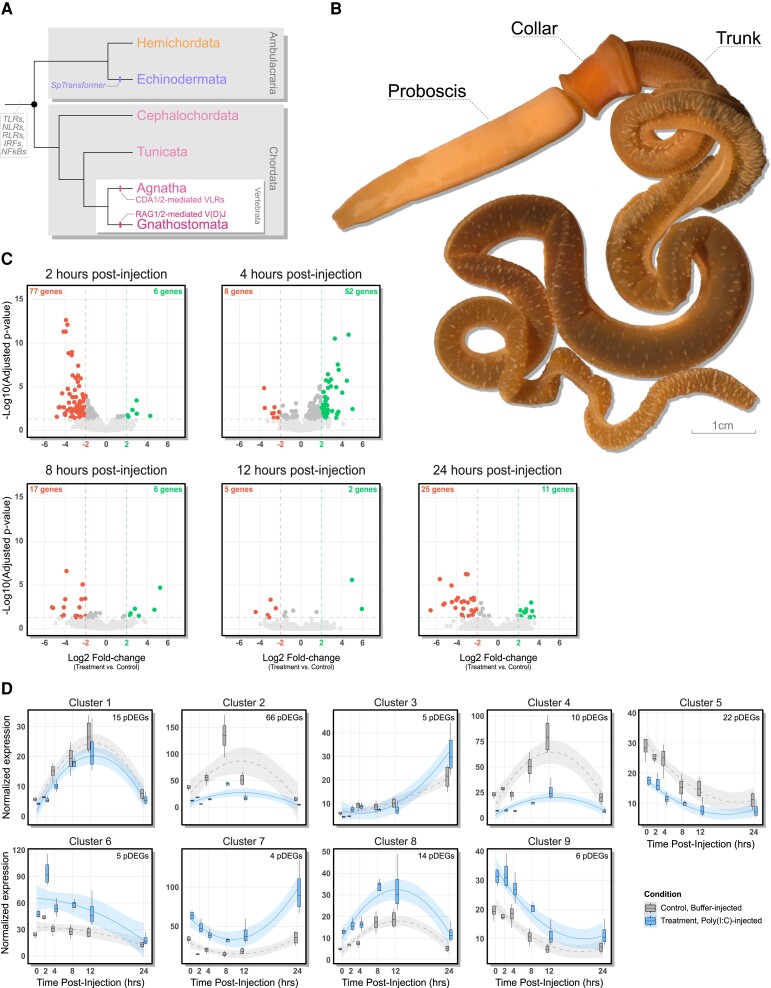
(*A*) Deuterostome phylogeny annotated for the evolution of key immunity pathways. The dashed line at the root denotes shared innate immunity pathways that were present in the last common ancestor of deuterostomes, and terminal branches are annotated for immunological novelties (see Loker 2012). Topology based on Li, Shen, et al. (2021). (*B*) Morphology of the acorn worm, *Saccoglossus kowalevskii*. (*C*) Volcano plots of DGE in poly(I:C)-injected worms relative to controls at each time point. Each point represents a gene; the horizontal dashed line represents the FDR-adjusted *P*-value cutoff (0.05) for statistical significance, and the vertical dashed lines are log_2_ fold-change thresholds set to |2|. Colored points represent genes that are above the significance threshold and exhibit log_2_ fold-change ≥ |2| expression. (*D*) Representative expression curves for each time-series differential expression analysis cluster. The dotted lines represent quadratic polynomial regression curves fit to the median gene expression within each cluster, and the shading shows 95% confidence intervals. The box plots show median expression variance across samples per group (*n* = 4 per group).

Evolutionary analyses of genome-scale data have shown that genetic components responsible for pathogen recognition and transcriptional regulation of immunity effectors are broadly conserved across Deuterostomia (fig. 1*A*; Tassia et al. 2021). Although comparative molecular evolution studies provide invaluable evidence on the origins of immune gene diversity, only a few invertebrate deuterostomes have been the subject of functional studies statistically powered for directly linking genes to immune response (Guo and Li 2021). The lack of a broad taxonomic representation in immunity research limits inferences concerning the ancestry of vertebrate immunity and may also misrepresent the diversity of immune systems that exist across deuterostomes. Furthermore, descriptive investigations aimed at identifying the unique assemblage of immunity genes encoded within genomic data are often constrained to be interpreted through the lens of model systems (e.g., human, mouse, and fly), thus potentially underestimating the true diversity of immunity mechanisms present across Metazoa (Dunn and Ryan 2015; David et al. 2019; Tassia et al. 2021).

Experiments leveraging RNA-seq for differential gene expression (DGE) analyses have provided valuable insights into the deployment of antiviral immune factors in echinoderms (Fuess et al. 2015; Ruiz-Ramos et al. 2020; Wu et al. 2020), the clade of invertebrate deuterostomes that have received the most attention with respect to viral disease ecology and immunology (Hewson et al. 2014; Miner et al. 2018; Guo and Li 2021). Within echinoderms, viruses have implicated as the infectious agent behind sea star wasting disease (Schiebelhut et al. 2018) and several fatal diseases in the sea cucumber, *Apostichopus japonicus* (an aquaculture species of economic importance; Zhang et al. 2015; Guo and Li 2021). As such, genetic investigations in echinoderms not only provide important phylogenetic context for understanding deuterostome immunity evolution, but also offer a genetic foundation upon which targeted molecular tools can be developed for conservation and aquaculture (Potts et al. 2021). Strikingly, recent virus-stimulated DGE analyses have revealed that echinoderm immunity utilizes many genes that are absent from current annotation databases, with only 1,183 (31.7% of total) differentially expressed genes (DEGs) in the sea star, *Pisaster ochraceus*, receiving high-confidence SwissProt annotations (Fuess et al. 2015), and 1,180 (67.5% of total) DEGs in *Holothuria leucospilota* annotated with at least a single gene ontology (GO) term (Wu et al. 2020). Such results are challenging to interpret without additional phylogenetic comparisons, as it remains ambiguous whether failure of annotation is driven by genetic innovation (and/or sequence divergence) among invertebrate lineages or due to loss of an ancestral antiviral toolkit in vertebrates. Together, these challenges highlight the importance of diverse taxonomic representation in comparative immunology and genomic studies (David et al. 2019).

As sister phylum to echinoderms (Halanych 1995; Cannon et al. 2014; Li, Shen, et al. 2021), hemichordates play an instrumental role in elucidating the ancestry of deuterostome traits, particularly in the field of evolutionary developmental biology (Lowe 2021). However, very little is known about hemichordate immunity. The immune system of hemichordates received some attention in the 1980s when the acorn worm hemichordate, *Saccoglossus horsti*, was shown to possess phagocytes within the collar and trunk coelomic cavities (fig. 1*B*) that are responsive to in vitro bacterial infection (Rhodes and Ratcliffe 1983), and the whole-body homogenate of the congeneric species, *Saccoglossus ruber*, exhibits strong antibacterial properties (Millar and Ratcliffe 1987). More recently, comparative genomic and transcriptomic studies have revealed that hemichordate genomes encode several major innate immunity protein families, including Toll-like receptors (TLRs) and their canonical pathway (Tassia et al. 2017; Chou et al. 2023), NOD-like receptors, RIG-I-like receptors (RLRs), interferon regulatory factors (IRFs), and nuclear factor κB (NF-κB) family members (Tassia et al. 2021). Furthermore, phylogenetic reconstruction of deuterostome TLRs shows hemichordates inherited an ortholog to TLR3, the TLR family member responsible for recognizing viral double-stranded RNA (dsRNA) in mammals (Tassia et al. 2017; Liu et al. 2020). Interestingly, type-I interferons, a group of secreted signaling molecules central to antiviral immunity, are hypothesized to be a vertebrate novelty, as no clear homologs have been identified in hemichordates or any other invertebrate phylum (Majzoub et al. 2019). Considering that the molecular components that are canonically upstream of interferon transcription are conserved in hemichordates (i.e., TLR3, RLRs, and IRFs), the genetic elements involved in hemichordate antiviral immunity have remained a provocative and open question.

Here, we induce and describe the transcriptomic response to dsRNA immune challenge in the acorn worm, *Saccoglossus kowalevskii* (fig. 1*B*), and interpret our findings in a comparative evolutionary framework. An acute viral infection was emulated within the proboscis cavity (fig. 1*B*) by injecting *S. kowalevskii* individuals with the synthetic viral dsRNA analog poly(I:C), a potent ligand of TLR3 and RLRs known to elicit an antiviral immune response within vertebrates (Kaur et al. 2019). Transcriptomes of individuals injected with solubilized poly(I:C) were compared against those that received injection buffer—directly accounting for transcriptional changes caused by wound healing (see Materials and Methods). For each experimental replicate (*n* = 4), adult female worms were sacrificed at 0, 2, 4, 8, 12, and 24 h postinjection (hpi), and sequencing libraries were independently constructed for each of the three body regions (i.e., proboscis, collar, and trunk) per worm (fig. 1*B*). In total, 144 RNA expression libraries were sequenced for our study (4 experimental replicates, 2 experimental conditions per replicate, 6 time points per condition, and 3 body regions per worm; supplementary table S1, Supplementary Material online).

## Results

*Saccoglossus kowalevskii* possesses an immune system capable of recognizing and transcriptionally reacting to poly(I:C) (fig. 1*C*). Using DESeq2 (Love et al. 2014) to test for DGE in poly(I:C)-injected worms relative to controls at each discrete time point, we resolved 423 unique DEGs (FDR-adjusted *P*-value ≤0.05, Wald test), including 193 genes exhibiting a log_2_ fold-change ≥ |2| (fig. 1*C*). Tissue was included as a covariate in the design formula for DESeq2 to account for tissue-dependent gene expression variation (supplementary fig. S1, Supplementary Material online); as such, DEGs resolved here reflect *S. kowalevskii*'s systemic transcriptional response to dsRNA infection. The quantity of DEGs was greatest at the two earliest time points, 2 hpi (138 DEGs) and 4 hpi (217 DEGs), with gene expression primarily reduced at 2 hpi (126/138 DEGs, 91.3%) and increased at 4 hpi (172/217 DEGs, 79.2%) in response to poly(I:C). At time points following 4 hpi, transcriptional effects were comparatively subtle (fig. 1*C*; supplementary fig. S2, Supplementary Material online), with 36, 12, and 43 DEGs resolved at 8, 12, and 24 hpi, respectively. A total of 83 DEGs at 2 hpi (60.1%), 60 DEGs at 4 hpi (27.6%), 23 DEGs at 8 hpi (63.9%), 7 DEGs at 12 hpi (58.3%), and 36 DEGs at 24 hpi (83.7%) exhibited a log_2_ fold-change in expression ≥ |2|.

We next applied MaSigPro (Nueda et al. 2014) to identify genes with altered temporal expression profiles in poly(I:C)-injected worms, thereby extending our differential expression analysis to capture regulatory trends over the whole time course experiment. Given patterns of gene expression at each independent time point (fig. 1*C*; supplementary fig. S2, Supplementary Material online), we applied a quadratic regression model to fit time-dependent gene expression curves and isolated 147 genes (hereby referred to as “profile DEGs” or pDEGs) with differences in temporal expression patterns following poly(I:C) injection (MaSigPro stepwise regression *R*-squared cutoff = 0.7). These 147 pDEGs fell into 9 clusters (see Materials and Methods), where each cluster represents a set of genes with correlated time-dependent expression curves (fig. 1*D*; supplementary figs. S3–S11, Supplementary Material online) and reflects potentially linked regulatory controls. Intersection between DESeq2 and MaSigPro results yielded only seven genes that were identified as differentially expressed in both analyses (supplementary Data, Supplementary Material online); however, these two data sets are not expected to overlap substantially as the underlying regression models test fundamentally different hypotheses of differential expression.

To provide biological context to these results, we assigned functional classifications via PANTHER (Thomas et al. 2003; Mi, Muruganujan, Ebert, et al. 2019), biological process GO (Ashburner et al. 2000; Gene Ontology Consortium 2021), KEGG Ontology (KO; Aramaki et al. 2020), and Pfam domain annotation (Mistry et al. 2021;supplementary Data, Supplementary Material online). Although both KO and Pfam annotations leverage profile hidden Markov models to account for evolutionary distance between query and subject sequences (see Functional Annotation section in Materials and Methods for details), PANTHER and GO annotations instead rely on directly intersecting gene/protein IDs with functional metadata contained within member databases (Mi, Muruganujan, Huang, et al. 2019). To accommodate the evolutionary distance between *S. kowalevskii* and the representative species in the PANTHER proteome database (Mi, Muruganujan, Ebert, et al. 2019), we used OrthoFinder (Emms and Kelly 2019) to infer hierarchical orthology groups (HOGs) for all genes in the *S. kowalevskii* genome and assign annotation-compatible gene IDs. Notably, only 179 of the 423 DEGs (42.3%) and 35 of the 147 pDEGs (23.8%) could be confidently assigned ortholog IDs. If gene identity was instead set using sequence similarity to SwissProt accessions (Buchfink et al. 2021; UniProt Consortium 2021), 298 DEGs (70.4%) and 78 pDEGs (53.1%) could be assigned annotation-compatible IDs. As OrthoFinder accounts for phylogenetic relationships between species and genes, PANTHER and GO-term enrichment were computed using ortholog IDs as a conservative estimation of functional enrichment (see supplementary fig. S12 and supplementary Data, Supplementary Material online for results from all enrichment analyses).

**Fig. 2.**
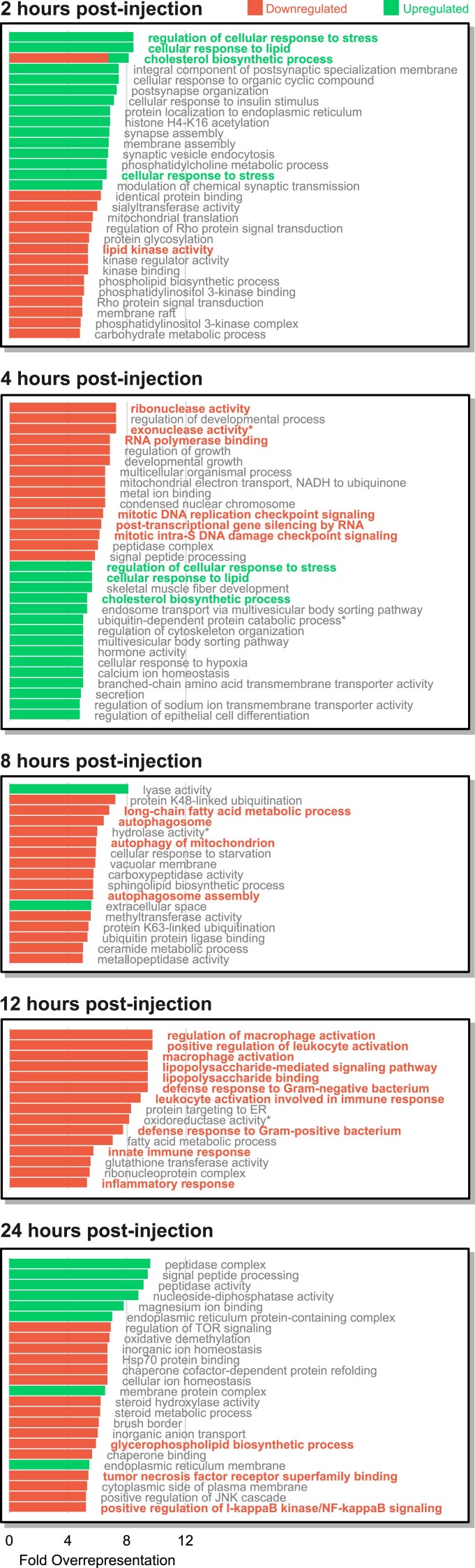
Top 15 biological process GO terms overrepresented among up- and downregulated genes at each time point. All terms shown here are statistically significant following a Pearson's *χ*^2^ test (Bonferroni-corrected *P*-value ≤ 0.05) relative to each term's frequency in the *Saccoglossus kowalevskii* genome and ranked by their log_2_ fold-overrepresentation (where all ties are included). Bolded terms are discussed in the main text. Overlapping bars reflect GO terms enriched in both up- and downregulated sets. Asterisk (*) denotes annotation description shortened for visualization purposes.

Enrichment of functional terms was estimated relative to their frequency in the *S. kowalevskii* genome (Bonferroni-corrected *P*-values ≤ 0.05, Pearson's *χ*^2^ test; tables 1 and 2). Peptidoglycan recognition (K01446, PTHR11022), a core component of immunity against Gram-positive bacteria, was reduced at 2 hpi. In contrast, negative regulation of interferon signaling (PTHR11949), NF-κB activation (PTHR15249), and NF-κB deactivation (K04734, K05872) were all enriched at 4 hpi, reflecting dynamic changes in immune response regulation at this time point. DNA repair (GO:0000724) and replication (GO:0033314), nuclease activity (GO:0016796), and transcription (GO:0010467) were also enriched among DEGs with reduced expression at 4 hpi (fig. 2), suggesting the antiviral immune response in *S. kowalevskii* involves a period of suppression of endogenous nucleic acid processing. Although biological process GO-term enrichment analysis did not resolve statistically significant overrepresentation of immunity annotations at 2 or 4 hpi, cellular response to stress (GO:0033554), and lipid metabolism (e.g., GO:0006695) were identified as enriched at each time point with statistical significance (fig. 2). Latter experimental time points (i.e., 8, 12, and 24 hpi) were broadly associated with downregulation of lipid metabolism and immunity (fig. 2), including reduced expression in genes associated with autophagy (GO:0000045; 8 hpi), inflammatory response (GO:0006954; 12 hpi), macrophage activation (GO:0042116; 12 hpi), and NF-κB signaling (GO:0043123; 24 hpi). Additionally, expression of genes associated with lipopolysaccharide recognition (PTHR10504) and tumor necrosis factor signaling (GO:0033209, PTHR10131, K03172–K03175) was reduced at 12 and 24 hpi, respectively. Strikingly, 2 hpi was the only subset enriched for genes without GO-term annotation (table 1). Considering all time point, functional enrichment analyses suggest *S. kowalevskii*'s transcriptional response to poly(I:C) is a dynamic process that involves regulatory changes across diverse molecular pathways, consistent with studies in vertebrates (Wu et al. 2016; Majzoub et al. 2019).

**Table 1.** DESeq2 Results Summary and Annotation Enrichment.

Time Point (hpi)	DEGs^b^	Enriched Annotations (Downregulated, Upregulated)^a^				
		Biological Process GO	PANTHER Families	PANTHER Pathways	KEGG	Pfam
2	138	22^c^, 37	28^c^, 14	0, 0	14^c^, 5	28, 13
4	217	44, 68	40, 97	3^c^, 2	26, 63	43, 92
8	36	21, 2	25, 4	3, 0	10, 4	29, 9
12	12	17, 0	6, 2	0, 0	5, 1	12, 6
24	43	33, 8	16, 3	0, 2	16, 6	21, 8

a Bonferonni-corrected *P*-value ≤ 0.05, Pearson's *χ*^2^ test.

b FDR-adjusted *P*-value ≤ 0.05, Wald test.

c Includes count for enrichment of genes without focal annotation.

**Table 2.** MaSigPro Results Summary and Annotation Enrichment.

	pDEGs^b^	Enriched Annotations^a^				
		Biological Process GO	PANTHER Families	PANTHER Pathways	KEGG	Pfam
Cluster 1	15	7^c^	3	0	4	13
Cluster 2	66	15^c^	26	1^c^	14	27
Cluster 3	5	1^c^	0	0	0	0
Cluster 4	10	11	3	0	2	10
Cluster 5	22	7^c^	4	0	4	20
Cluster 6	5	3	4	0	1	2
Cluster 7	4	11	3	2	1	4
Cluster 8	14	7	3	3	1	10
Cluster 9	6	6	5	0	19	3

a Bonferonni-corrected *P*-value ≤ 0.05, Pearson's *χ*^2^ test.

b MaSigPro stepwise regression *R*-squared ≥ 0.70.

c Includes count for enrichment of genes without focal annotation.

**Fig. 3.**
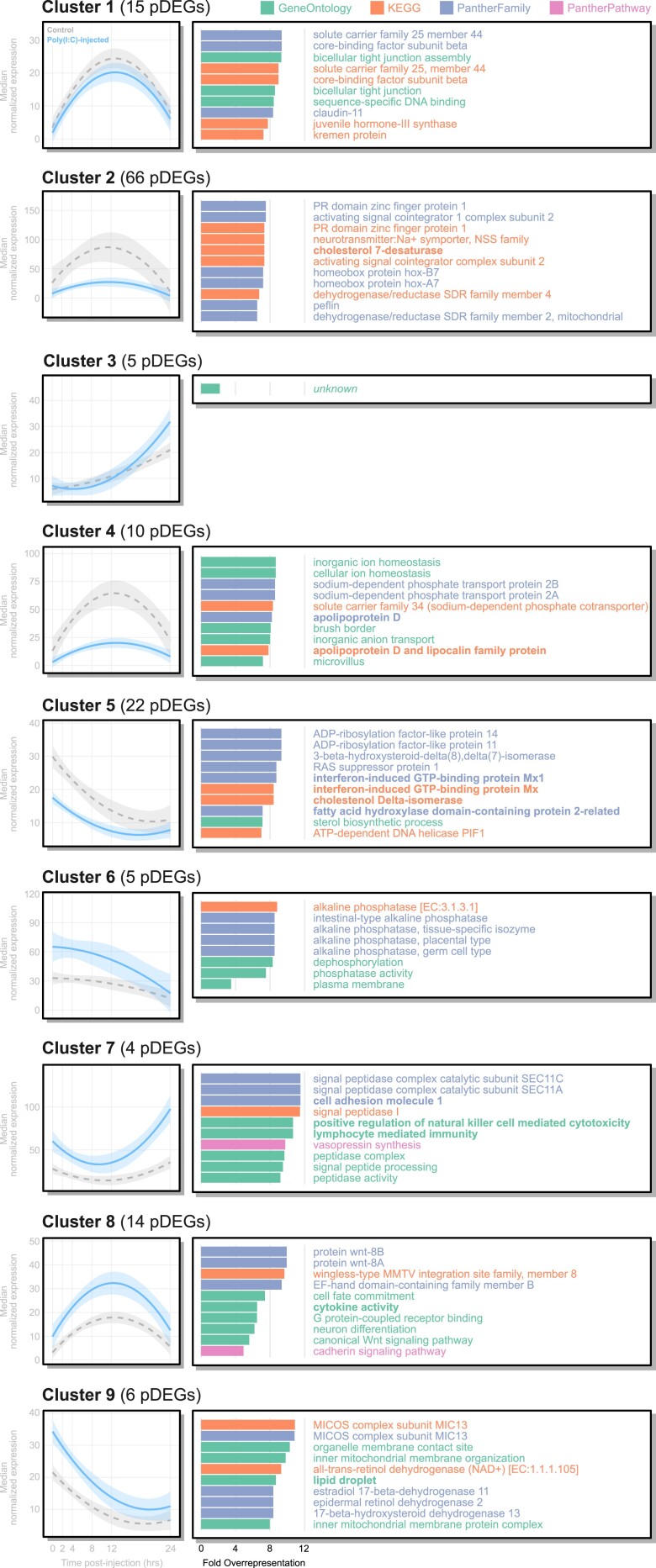
Top 10 overrepresented annotation terms in each pDEG cluster. All terms shown here are statistically significant following a Pearson's *χ*^2^ test (Bonferroni-corrected *P*-value ≤ 0.05) relative to each term's frequency in the *Saccoglossus kowalevskii* genome and ranked by their log_2_ fold-overrepresentation (where all ties were included). Left column shows median expression curves within each cluster. Bolded terms represent those discussed in the main text.

Functional enrichment among pDEG clusters was interpreted in the context of qualitative differences between expression profiles for poly(I:C)-injected and control worms (figs. 1*D* and 3). pDEG clusters 5, 7, and 8 were enriched for genes associated with immunity (fig. 3). Cluster 5 was enriched for terms associated with the antiviral protein Mx (PTHR11566, K14754), a peptide with broad antiviral properties and downstream of interferon signaling (Haller and Kochs 2020). As cluster 5 pDEGs exhibited trends of early expression reduction in poly(I:C)-injected worms followed by convergence with control expression levels (fig. 3; supplementary fig. S7, Supplementary Material online), this suggests Mx transcription is reduced early on during *S. kowalevskii*'s immune response against dsRNA. Cluster 7 is enriched for terms associated with *CADM1* (GO:0002449, GO:0045954), an immunoglobulin domain-containing protein with known roles in regulating apoptosis during tumor metastasis and Epstein–Barr virus transmission, and may be regulated by NF-κB signaling (Li, Gao, et al. 2021; Shirogane et al. 2021). Cluster 7 pDEG expression is higher in immune-challenged worms and diverges from controls at both early and late time points (fig. 3; supplementary fig. S9, Supplementary Material online). Cytokine activity (GO:0005125) was overrepresented in cluster 8, where challenged worms exhibited increased expression at intermediate time points relative to controls (fig. 3; supplementary fig. S10, Supplementary Material online). Finally, cluster 3 was only enriched for pDEGs without GO-term annotations and six Pfam domains (table 2), a striking result given that pDEGs in this cluster appear to only differ between experimental conditions at late time points (supplementary fig. S5, Supplementary Material online).

## Discussion

The acorn worm, *S. kowalevskii,* can mount a dynamic transcriptional response following injection of the potent viral dsRNA analog, poly(I:C) (fig. 1). Among genes implicated in *S. kowalevskii* antiviral immunity are a collection of orthologs associated with canonical immune response pathways (figs. 2 and 3; supplementary Data, Supplementary Material online), further fortifying their evolutionarily conserved role in metazoan immunity (Tassia et al. 2021). Consistent with DGE studies in echinoderms (Fuess et al. 2015; Wu et al. 2020), we also recovered many DEGs that could not be confidently assigned orthology to genes in biomedical model species (supplementary Data, Supplementary Material online), limiting functional inference. Although genes with unresolved functional annotations were represented among DEGs at each time point, we only detected their enrichment at 2 hpi. This finding has important implications for understanding immunity evolution in deuterostomes, as their combined enrichment and lack of annotation suggest these genes may be involved in the early antiviral response of hemichordates and/or invertebrate deuterostomes (fig. 1*A*).

Among vertebrates, dsRNA viruses are recognized by TLR3 and RLRs, a subset of the innate immune system's suite of pattern recognition receptors (Li and Wu 2021). Binding of dsRNA by TLR3 and RLRs is canonically associated with the activation of IRFs and NF-κB, respectively, transcription factors responsible for the induction of proinflammatory cytokine expression which initiate pleiotropic effects such as immune cell recruitment and apoptosis (Mogensen and Paludan 2001). NF-κB and IRF signaling were enriched among genes with reduced expression at 4 hpi, suggesting systemic deactivation of these pathways early in our experiment. Both vertebrate NF-κB and IRF signaling act in compensatory regulatory feedback loops which reduce their own expression over time to avoid harmful overstimulation (Blach-Olszewska and Leszek 2007; Dorrington and Fraser 2019; Andresen et al. 2020). Furthermore, targeted expression studies have shown that NF-κB and IRF transcription follows a similar trend in urchin larvae following poly(I:C) exposure (Pancer et al. 1999; Chiaramonte et al. 2019). In light of this evidence, the reduced expression of genes associated with these pathways at 4 hpi suggests NF-κB and IRF activation occurred at an earlier unsampled time point in our experiment. This hypothesis is consistent with enrichment trends at latter time points (i.e., 8, 12, and 24 hpi) which showed reduced expression of genes associated with macrophage activation and TNFR signaling—mechanisms canonically downstream of NF-κB and IRF signaling (Banete et al. 2021). Although additional time points and experimental manipulation are required to confidently resolve the temporal dynamics of NF-κB and IRF transcription in *S. kowalevskii*, our results suggest hemichordates, echinoderms, and chordates inherited a shared NF-κB/IRF regulatory circuit from their last common ancestor.

Among the subset of *S. kowalevskii* genes that could be annotated for function (figs. 2 and 3; tables 1 and 2), several patterns emerged that were consistent with the described antiviral immune responses in mammals. Beginning at our earliest time points (and present in pDEG clusters 2, 4, 5, and 9; fig. 3), we observed increased expression of multiple fatty acid synthesis genes (fig. 2). In mammals, fatty acid synthesis following viral infection is stimulated by interferon signaling and is hypothesized to construct a hostile cellular environment for viruses (Wu et al. 2016). Increased synthesis has also been associated with the host's increased demand for fatty acids during cellular proliferation and production of inflammatory lipid mediators (Serhan et al. 2014; Bai and Li 2019). Our results implicate lipid metabolism in *S. kowalevskii*'s response to viral stimulus, suggesting the regulatory interaction between immunity and fatty acid synthesis may have been present in the last common ancestor of deuterostomes. We also observed reduced expression in genes associated with nucleic acid processing at 4 hpi. Similar associations have been observed in murine-derived dendritic cells (Olex et al. 2016), which were hypothesized to correlate with a shift in energy allocation during immune response. Speculatively, reduced nucleic acid processing may reflect an antiviral mechanism in which DNA/RNA processing machinery is suppressed to inhibit viral replication. Nonetheless, differential expression of lipid metabolism and nucleic acid processing genes suggest the broader metabolic landscape of immunity may have been inherited alongside canonical innate immunity signaling pathways (Tassia et al. 2017, 2021; Chou et al. 2023).

By directly accounting for tissue-specific transcriptional effects in our experimental design, DEGs identified in our study reflect *S. kowalevskii*'s systemic response to acute viral infection. This distinction is important, as discrete hematopoietic and immunocyte sources are yet to be identified among hemichordates (Tassia et al. 2018); thus, the fine-scale transcriptional response mounted by *S. kowalevskii* to dsRNA infections remains unclear. Within the evolutionary context of Ambulacraria (fig. 1*A*), immunological studies in purple urchin have primarily focused on larval immunity, making direct comparisons between our study in adult acorn worms and those in larval echinoderms challenging. Importantly, urchin larval immunity remains one of the most well-characterized immunological systems among invertebrates (Buckley and Rast 2019). With the recent development of experimentally tractable hemichordate larvae in the acorn worm *Schizocardium californicum* (Gonzalez et al. 2018; Bump et al. 2022), direct comparisons of immune responses between homologous developmental time points will be invaluable for testing hypotheses on the ancestry of immune cell recruitment, inflammatory response, and NF-κB/IRF signaling (Ho et al. 2016; Chiaramonte et al. 2019).

In our study, we present evidence that *S. kowalevskii*'s transcriptional response to dsRNA infections exploits regulatory changes in both canonical innate immunity pathways and those auxiliary to the immune response, such as lipid metabolism and nucleic acid processing (figs. 2 and 3). By conservatively estimating orthology across the *S. kowalevskii* genome, we also observe statistical enrichment of genes without GO annotations at 2 hpi and in pDEG clusters 1, 2, 3, and 5 (tables 1 and 2), which may collectively represent evolutionarily divergent genes associated with immunity signaling pathways—consistent with similar findings in echinoderms (Fuess et al. 2015; Wu et al. 2020). Importantly, our results support the hypothesis that modern deuterostome phyla inherited a complex innate immunity signaling toolkit from their last common ancestor, a conclusion consistent with differential expression studies performed across the superphylum (Tassia et al. 2017, 2021; Chou et al. 2023). This hypothesis, while still warranting further controlled experimental evidence (ideally incorporating multiple directly comparable deuterostome lineages under a shared experimental framework), provides the foundation for elucidating the evolution of immunity across Deuterostomia.

## Materials and Methods

### Animal Handling

Worms were obtained intertidally from Waquoit Bay, MA in September 2018 and 2019. Detailed information on *S. kowalevskii* collection and general upkeep can be found in (Lowe et al. 2004). All worms were females and had spawned at least 4 days prior to treatment. Immediately prior to injection, individual worms were placed into a single well of a six-well plate containing filtered seawater, and each plate was placed on ice to relax worms prior to injection. During injection, individuals were temporarily submerged in a 3.75% solution of magnesium chloride (dissolved in filtered seawater) to inhibit muscle contraction. Following injection, worms were replaced to their six-well plates and placed into a flow-through seawater table until their incubation time was complete.

### Experimental Design

Two experimental replicates were performed in both September 2018 and September 2019. Individual worms were injected with either 1) an agonist solution containing 10 μg/ml high- and low-molecular weight poly(I:C) (tlrl-pic and tlrl-picw, respectively; InvivoGen) dissolved in an injection buffer containing 1× phosphate-buffered saline (10 mM sodium phosphate, 0.15 mM sodium chloride) and 5 mg/ml Calceine fluorescent dye, or 2) injection buffer alone. Six time points were used for our study: 0, 2, 4, 8, 12, and 24 hpi. After incubation, individuals were dissected into proboscis, collar, and trunk before being flash-frozen in liquid nitrogen and stored at −80 °C. For each experimental replicate, 36 samples were generated (2 conditions, 6 time points, and 3 body regions). In total, 144 samples were collected and prepared for sequencing (supplementary table S1, Supplementary Material online).

Needles were pulled from Sutter Instruments Co.'s thin-wall borosilicate glass with filament (BF100-78-10) on a Sutter Instruments Co.'s Model P-97 (parameters: P = 200, Heat = 520, Pull = 60, Vel = 100, Time = 175). Needles were loaded with 1 μl of injection solution (as described above), and the injection was propelled by nitrogen gas using a MPPI-3 Pressure Injector (Applied Scientific Instrumentation, Inc.). Worms were injected into the posterior proboscis directly anterior to the proboscis neck.

### Library Preparation and Sequencing

RNA extractions were performed using the Qiagen RNeasy Mini Kit (Qiagen) and on-column RNase-Free DNase Set (Qiagen). RNA concentration and purity were assessed on a TapeStation 2200 (Agilent Technologies, Inc.). Sequencing libraries were constructed with the QuantSeq 3′ mRNA-Seq Library Prep Kit FWD for Illumina (Lexogen) in conjunction with the i5 6 nt Dual Indexing Add-on Kit (Lexogen). Optimum amplification cycles per library were assessed using the PCR Add-on Kit for Illumina (Lexogen). Libraries were sent to the HudsonAlpha Institute for Biotechnology (Huntsville, AL, USA), where they were sequenced on a NovaSeq (single-end, 100 bp; Illumina, Inc.). Sequencing statistics are reported in supplementary table S1, Supplementary Material online.

### Sequence Preprocessing

All 144 sequence read libraries were cleaned using *fastp* (version 0.19.7; Chen et al. 2018) to remove adapter sequences (AGATCGGAAGAGCACACGTCTGAACTCCAGTCA), 3′ polyX tails, and perform per-base quality trimming using a “cut right” sliding window with default parameters.

Each library was mapped to the *S. kowalevskii* reference genome (version Skow_1.1; downloaded from the NCBI genome repository in December 2019) using STAR (version 2.7.0; Dobin et al. 2013). STAR parameters follow Lexogen's recommendations for the QuantSeq 3′ mRNA-Seq Library Prep Kit FWD for Illumina; mapping statistics can be seen in supplementary table S1, Supplementary Material online. Following read alignment, count matrices were obtained using HTSeq-count (version 0.11.2; Anders et al. 2015) with “intersection-nonempty” defined for handling reads which overlap multiple features, strandedness set to sense orientation, and both feature type and ID attribute set to “gene” for quantification. Read-map quantification was not normalized prior to DGE analyses.

### Differential Gene Expression Analysis

DESeq2 (version 1.30.1; Love et al. 2014) was used to identify DEGs between treatment (i.e., poly(I:C)-injected individuals) and control (i.e., buffer-injected individuals) at each time point using the following design formula:


$$\begin{array}{rl} & ∼Condition+Time\;Postinjection\;(TPI)+Tissue\\ & +\;Condition:TPI \end{array}$$

For Wald tests, log_2_ fold changes per gene were estimated using a linear contrast of *Condition + Condition:TPI* where reference levels were control individuals at the same time point. The false discovery rate adjusted *P*-value cutoff for DESeq2 was set to 0.05. *TPI* and *Tissue* were included as categorical covariates to capture expression variation derived of tissue-dependent (supplementary fig. S1, Supplementary Material online) and time-dependent effects that can be isolated from the treatment contrast (e.g., wound healing or biorhythms, respectively). Importantly, the overall experimental design of our study controls for several biological factors (i.e., sex and sexual maturity) that could otherwise influence the number of genes (and subsequent conclusions) associated with *S. kowalevskii*'s antiviral immune response. Plots describing gene expression dispersion, independent filtering cutoff inference, and Cook's distance outlier thresholding are presented in supplementary figures S13–S16 and table S2, Supplementary Material online.

In addition to DESeq2, MaSigPro (Nueda et al. 2014) was used to leverage time-series data and identify genes that exhibit differential expression curves between treatment and control conditions. Because MaSigPro cannot accept a covariate array, raw sequencing libraries for each of the three body regions per individual were first consolidated, and quantification was performed using the same HT-Seq protocol as described in Sequence Preprocessing section. Read counts per gene were loaded into DESeq2, and size-factor normalized read counts (accounting for library depth) were used as expression input for MaSigPro. Genes with mean read counts <10 in 3 or more sequence libraries were removed as low-expression outliers (Cardoso-Moreira et al. 2019). MaSigPro was run with a quadratic regression model using *condition* and *time postinjection* as predictor variables. Output clustering was performed using the “Mclust” algorithm (Scrucca et al. 2016) to estimate the optimum number of expression clusters, and hierarchical clustering was applied for data partitioning.

Data were visualized using ggplot2 (version 3.3.3; Wickham 2016). DESeq2 and ggplot2 were run within RStudio (version 2022.07.2 + 576, R Studio Team 2015; R version 4.2.0, R Core Team 2015).

### Functional Annotation

Each peptide sequence in the *S. kowalevskii* reference genome (Skow_1.1) protein data set was assigned a best-hit SwissProt accession (accessed January 2023; UniProt Consortium 2021) to 1) the top-scoring human accession (“SwissProt Human”), and 2) the top-scoring accession intersected with species represented in the PANTHER Reference Proteome dataset (“SwissProt PANTHER”) using DIAMOND (Buchfink et al. 2021). Top-scoring hits were ranked by the greatest bit score, lowest *e*-value, and highest percent of identical matches. In addition to SwissProt linear sequence similarity assignments, OrthoFinder (version 2.5.4; Emms and Kelly 2019) was run under default parameters with the following genomes: *S. kowalevskii* (Skow_1.1), human (GRCh38), mouse (GRCm39), zebrafish (GRCz11), *Caenorhabditis elegans* (WBcel235), and *Drosophila melanogaster* (Release 6 plus ISO1 MT). A third ID (“Orthofinder HOG”) was assigned to each *S. kowalevskii* protein with homologous human proteins falling within the same HOG. When multiple human paralogs were present within a HOG, the corresponding *S. kowalevskii* protein was assigned both gene symbols. All annotations referenced in the main text were derived from the “Orthofinder HOG” data set, given the method's documented performance for accurately inferring orthology relative to traditional sequence similarity methods (Emms and Kelly 2019). These three accession data sets (i.e., “SwissProt Human,” “SwissProt PANTHER,” and “Orthofinder HOG”) were processed with PANTHER's (version 17) gene list analysis tool to assign each PANTHER-compatible accession functional context by PANTHER Pathway, PANTHER Family, and GO-Slim (Gene Ontology Consortium 2021). Finally, each *S. kowalevskii* protein was annotated with KO using kofamKOALA (Aramaki et al. 2020) and best-fit Pfam domain(s) (Mistry et al. 2021) using HMMER (Eddy 2009) and the *Best_fit_domains.py* function from TIAMMAt (Tassia et al. 2021). An intersection of these annotation methods is presented in supplementary figure S12, Supplementary Material online.

### Functional Enrichment

Statistical enrichment for each annotation category above was calculated by performing a Pearson's *χ*^2^ test (Bonferroni-corrected *P*-value significant threshold = 0.05) on a contingency table constructed of each term's occurrences in the *S. kowalevskii* genome versus a focal subset (e.g., significantly upregulated 2 hpi or genes within an expression profile cluster as inferred by MaSigPro). Fold-overrepresentation of each functional term (*i*) was computed as log_2_(*d*(*O_i_*,*E_i_*)/*E_i_*), where *O* is the observed term count and *E* is the expected count asserted from the Pearson's *χ*^2^ null hypothesis.

## Supplementary Material

msad097_Supplementary_Data

## Data Availability

Raw sequence reads are publicly available via the NCBI accession PRJNA775633. Supplementary Data is available via Zenodo (10.5281/zenodo.7758058).
