# Supplementary material for: Induced Immune Reaction in the Acorn Worm, Saccoglossus kowalevskii, Informs the Evolution of Antiviral Immunity
Source: Mol Biol Evol. 2023 Apr 28;40(5):msad097. doi: 10.1093/molbev/msad097 (PMC10210618; doi:10.1093/molbev/msad097)
Supplement: msad097_Supplementary_Data [file msad097_supplementary_data.pdf]

Supplementary Information

Induced immune reaction in the acron worm, *Saccoglossus kowalevskii*, informs the evolution of antiviral immunity

Michael G. Tassia, Haley A. Hallowell, Damien S. Waits, Ryan C. Range, Christopher J. Lowe, Rita M. Graze, Elizabeth Hiltbold Schwartz, Kenneth M. Halanych

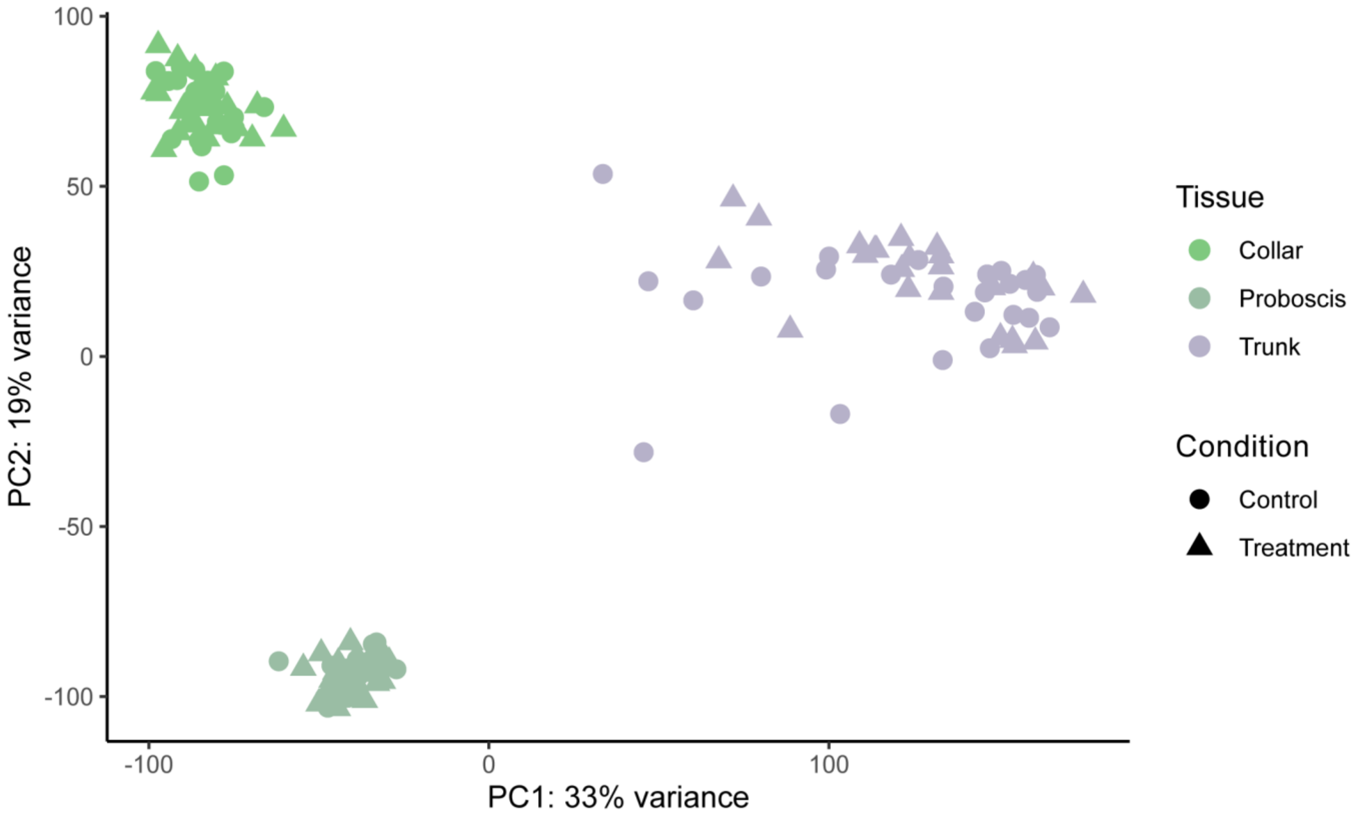

Figure S1. Principle component plot of the variance stabilized count matrix for DESeq2 experiment. Samples are colored by condition (treatment or control) and tissue (proboscis, collar, or trunk). Approximately 52% of variance can be attributed to differences in gene expression among the three tissue types, highlighting the importance of its inclusion as a covariate in our experimental design.

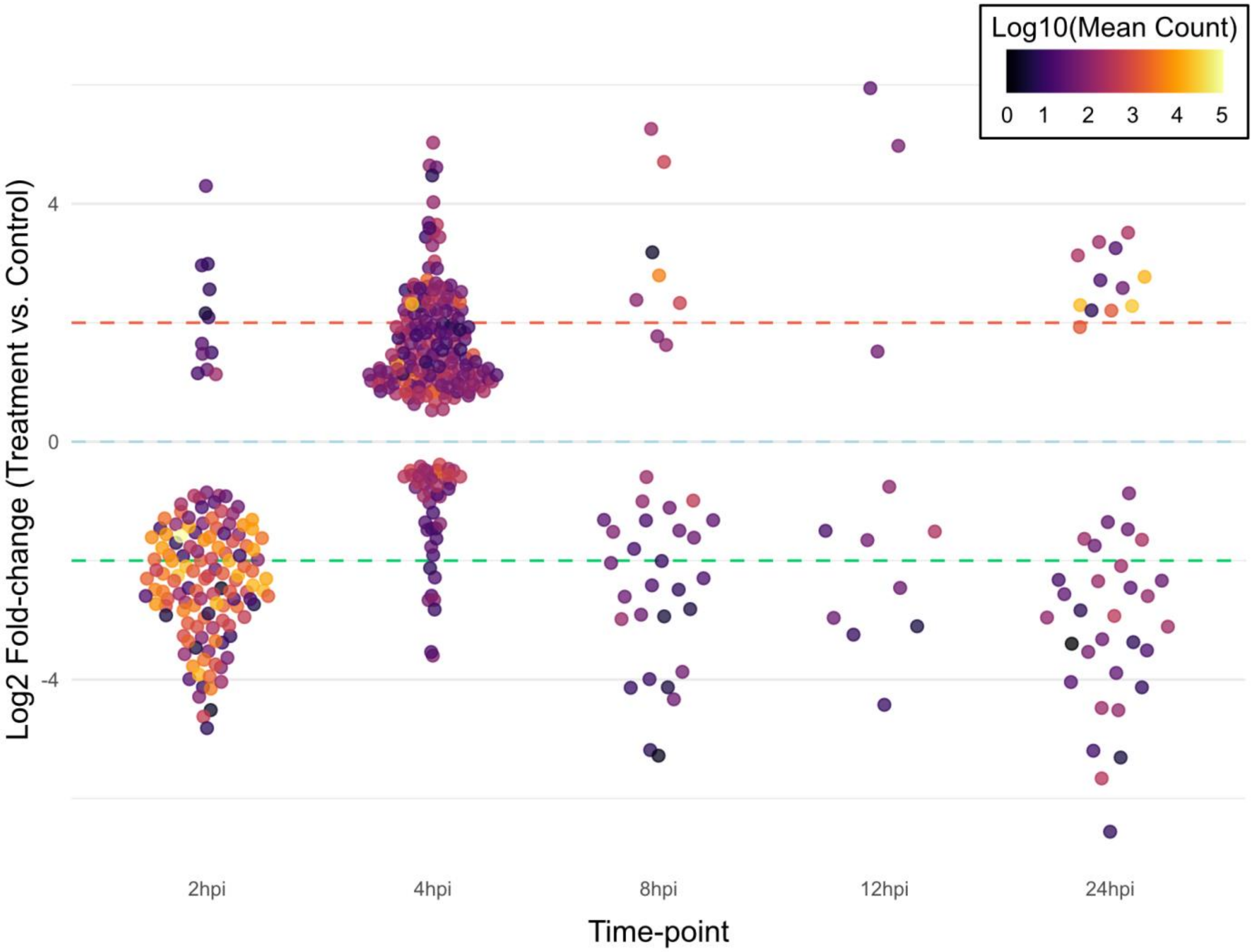

Figure S2. Distribution of Log2 fold-change estimate in gene expression per timepoint. Each point represents a gene (FDR-corrected p-value ≤ 0.05), and color reflects mean count estimations by DESeq2.

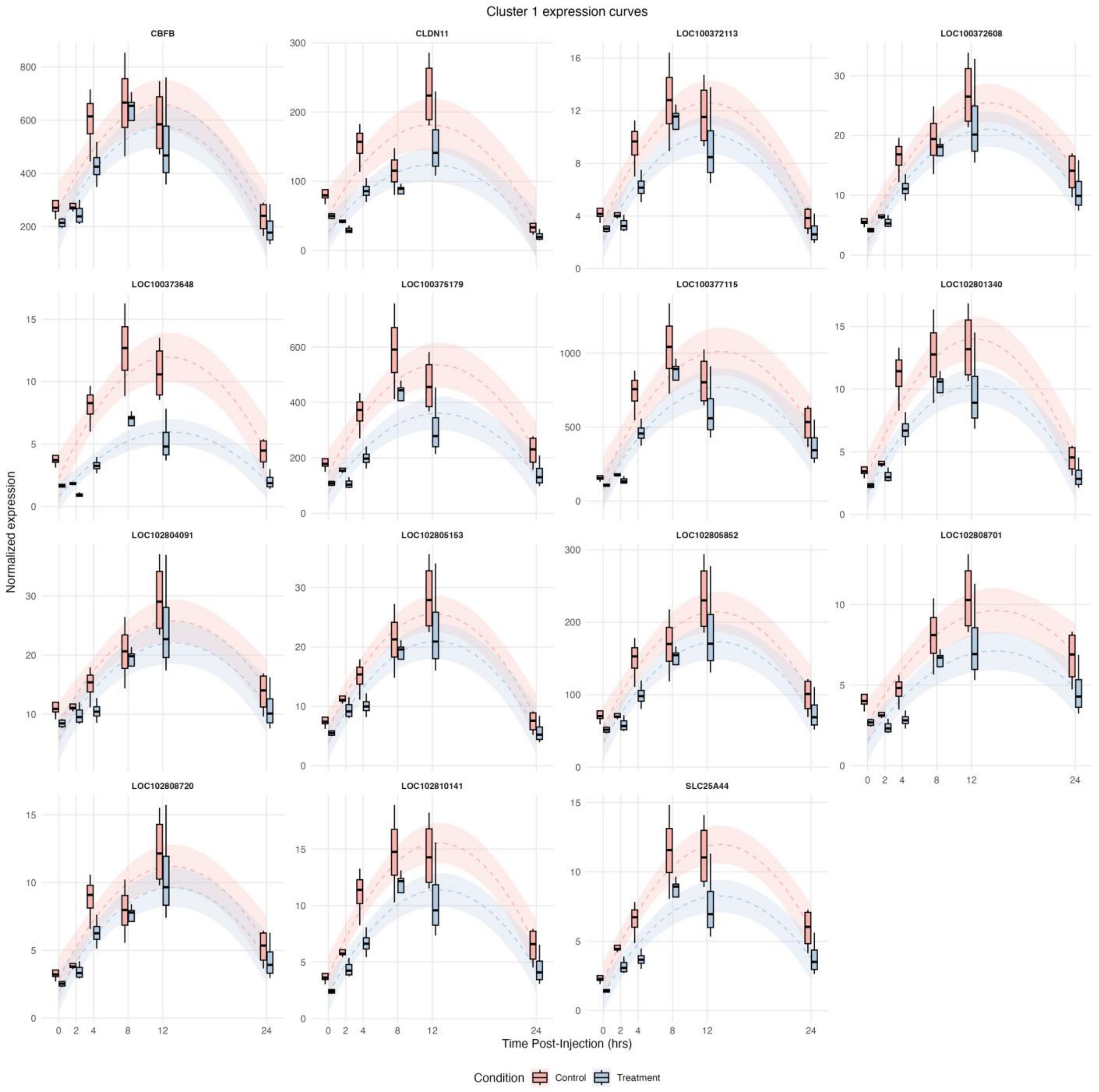

Figure S3. Distribution of quadratic regression curves fit to Cluster 1 pDEGs.

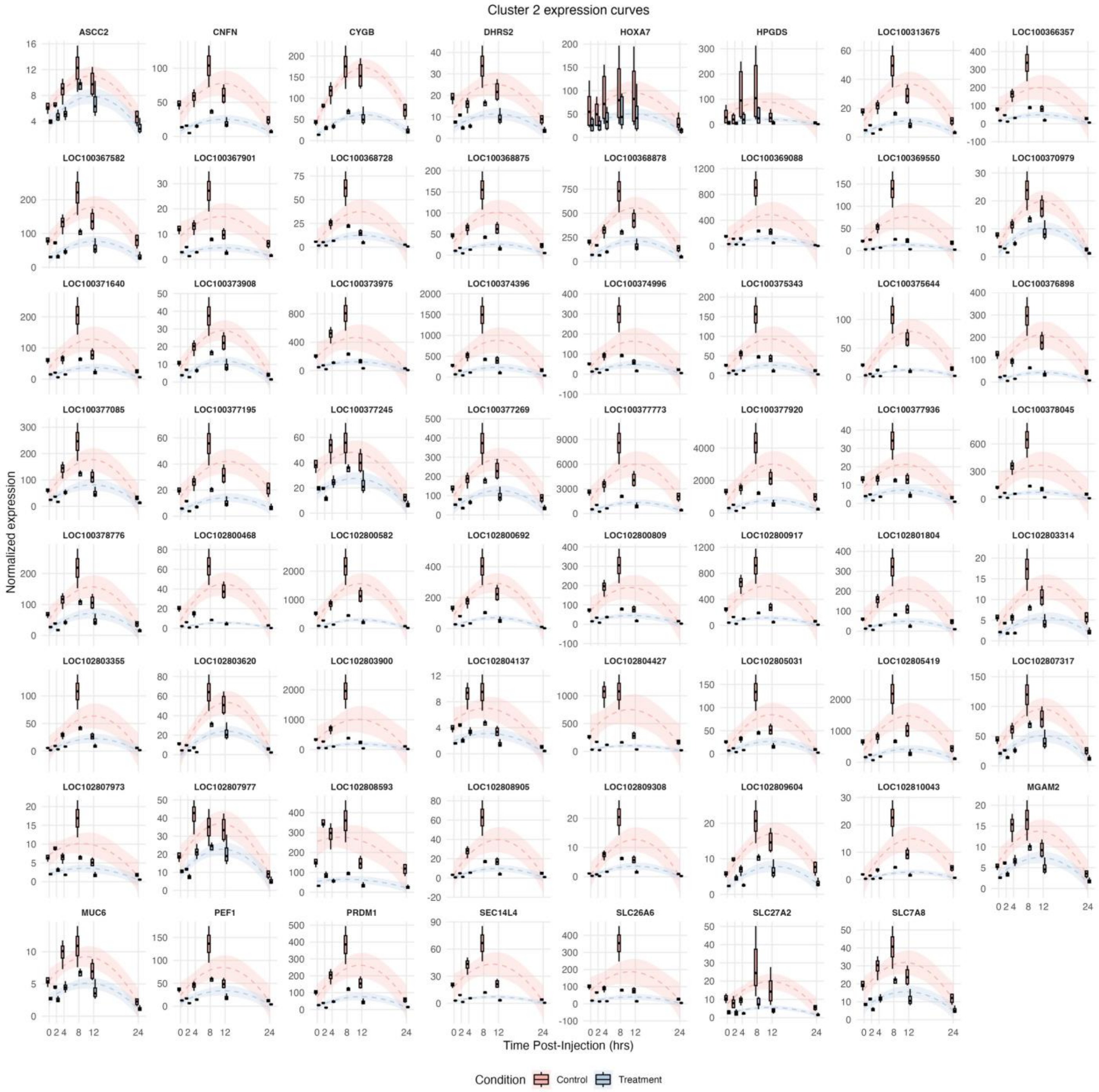

Figure S4. Distribution of quadratic regression curves fit to Cluster 2 pDEGs.

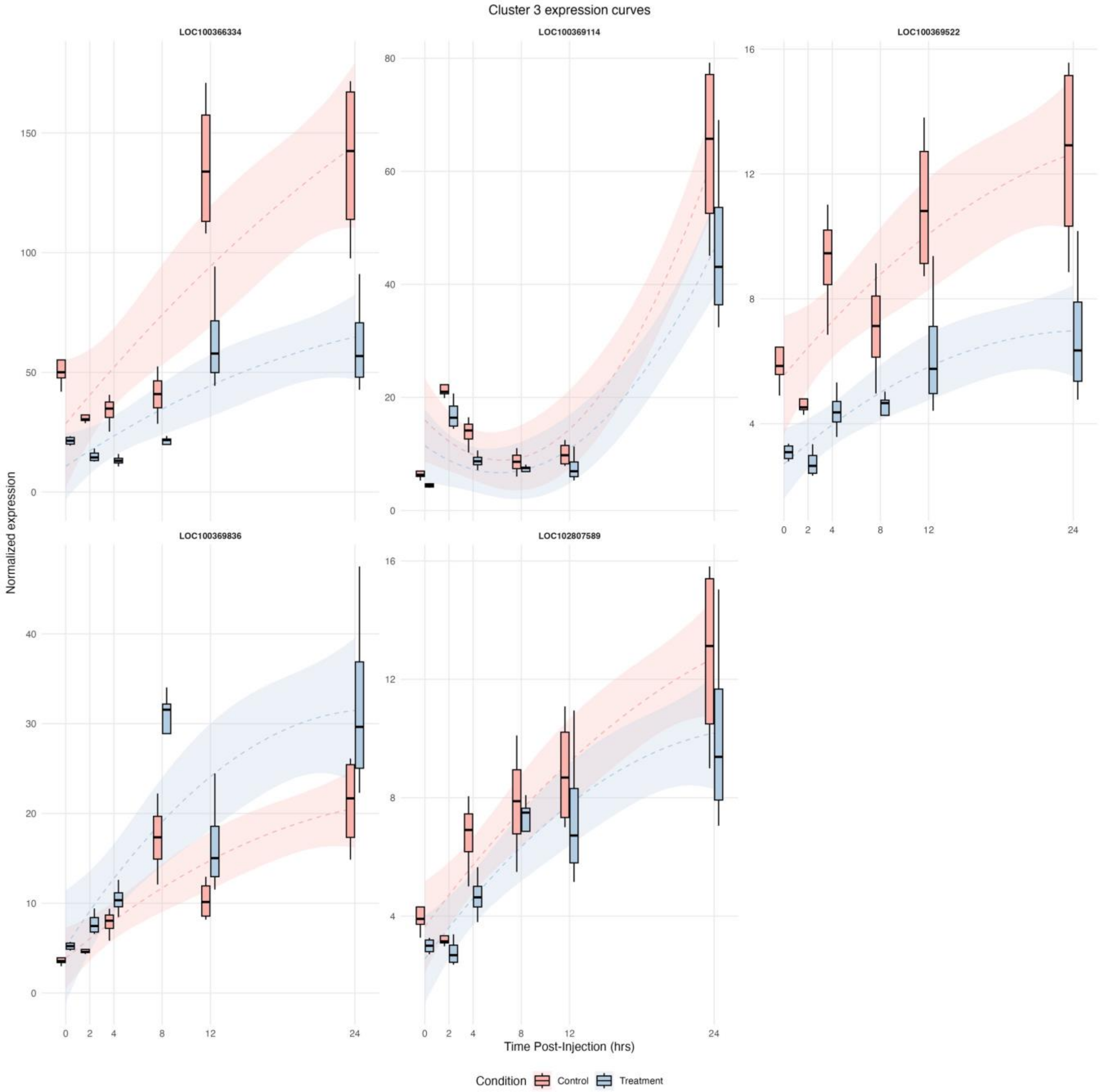

Figure S5. Distribution of quadratic regression curves fit to Cluster 3 pDEGs.

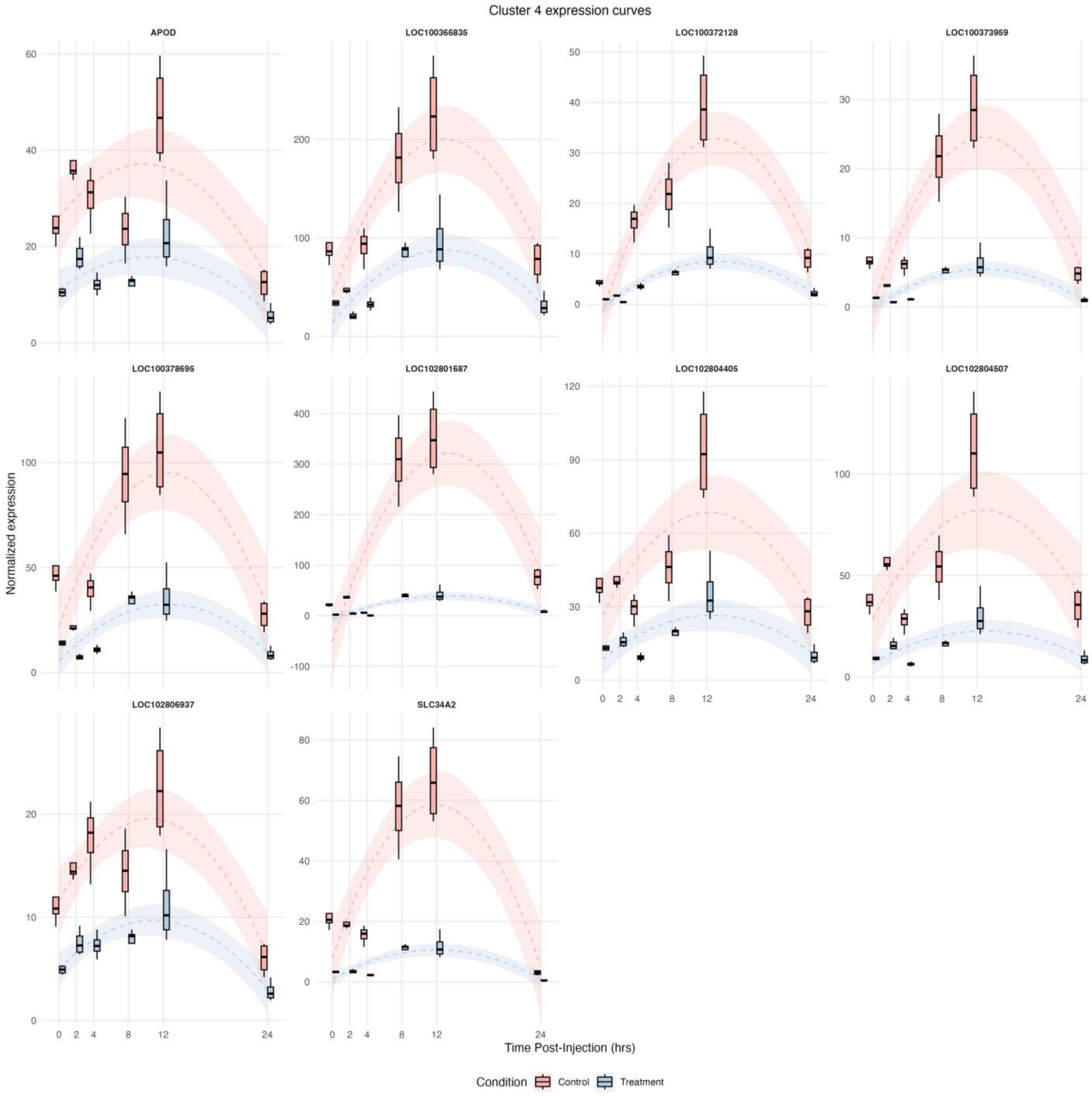

Figure S6. Distribution of quadratic regression curves fit to Cluster 4 pDEGs.

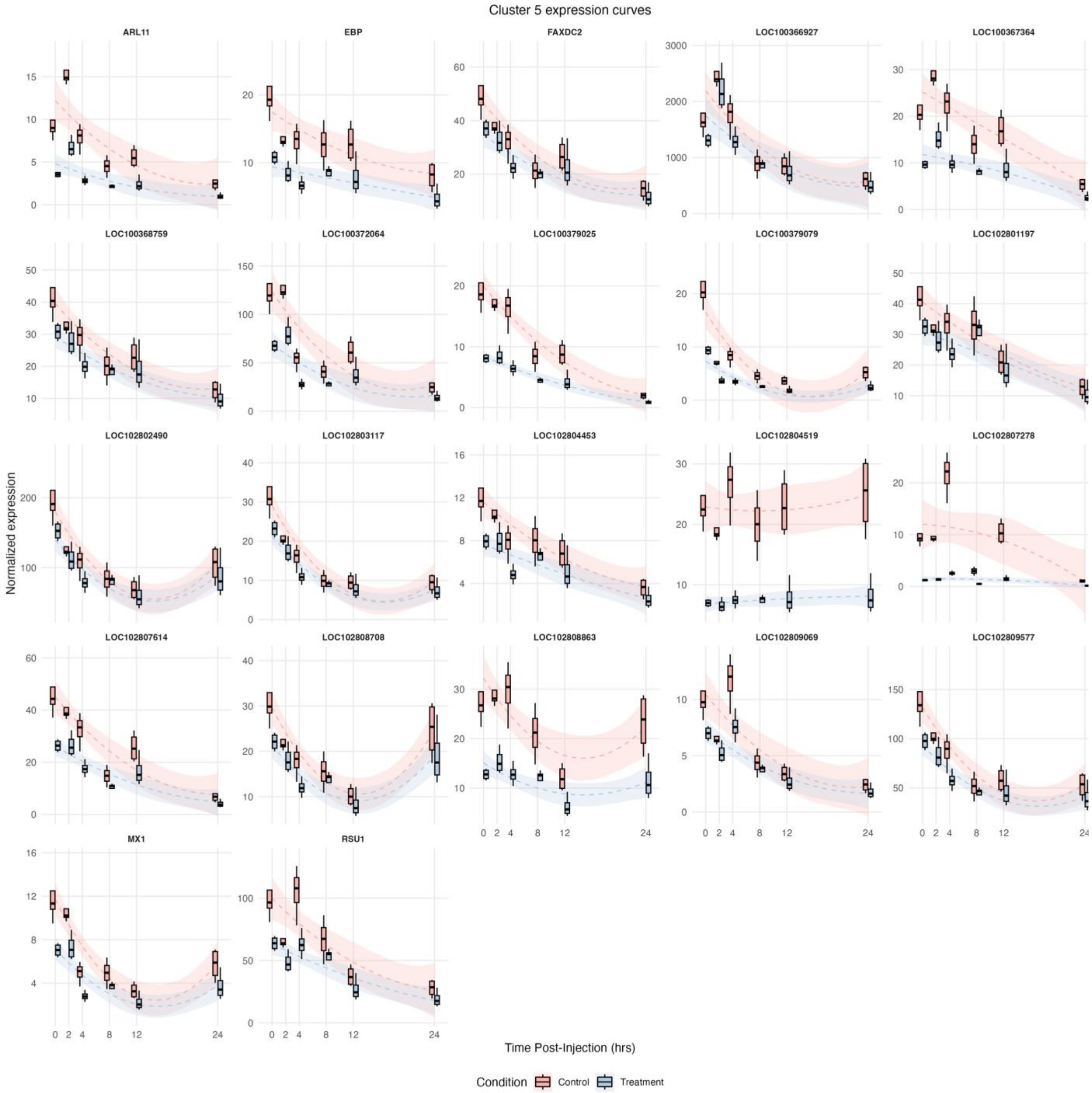

Figure S7. Distribution of quadratic regression curves fit to Cluster 5 pDEGs.

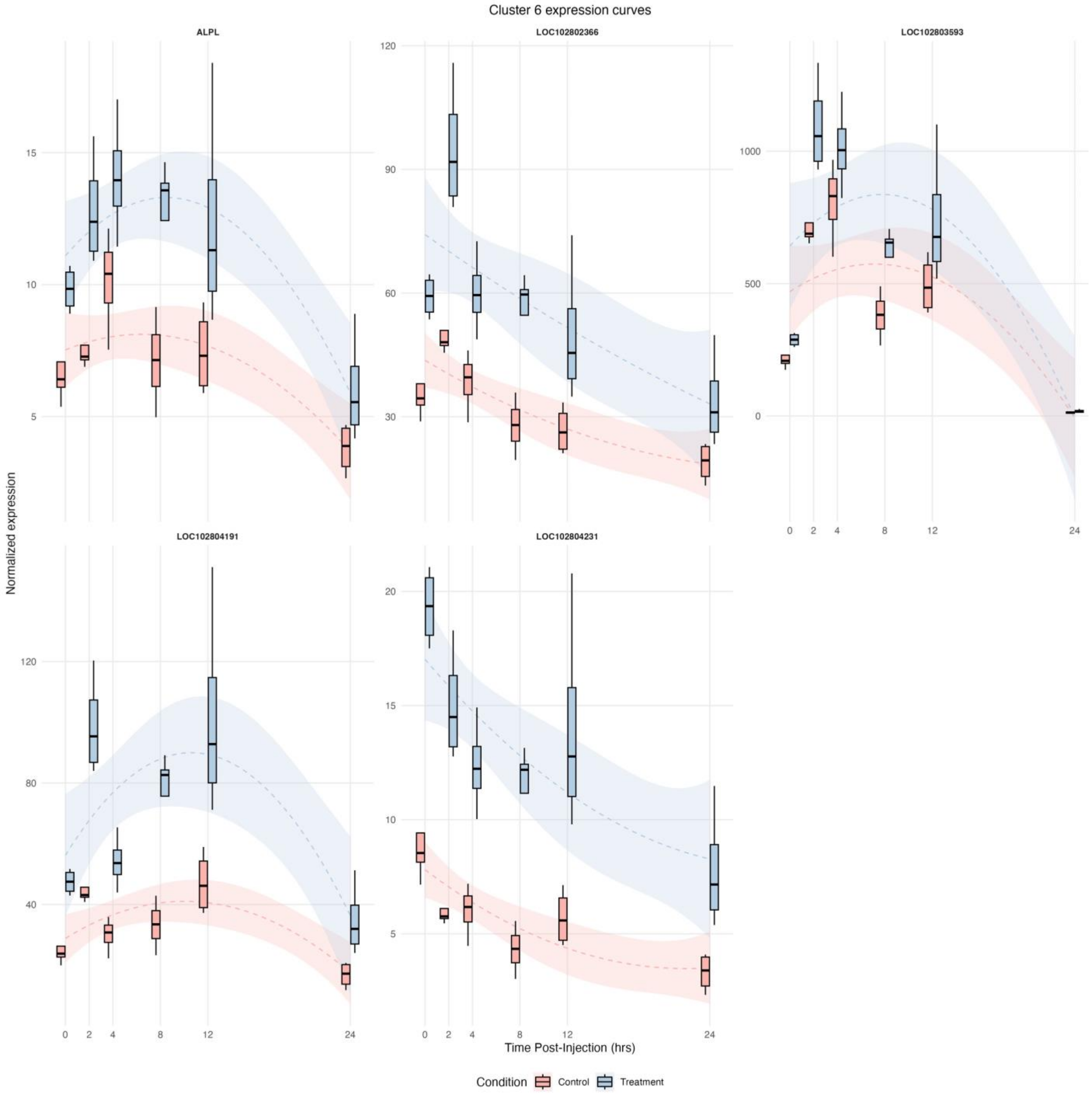

Figure S8. Distribution of quadratic regression curves fit to Cluster 6 pDEGs.

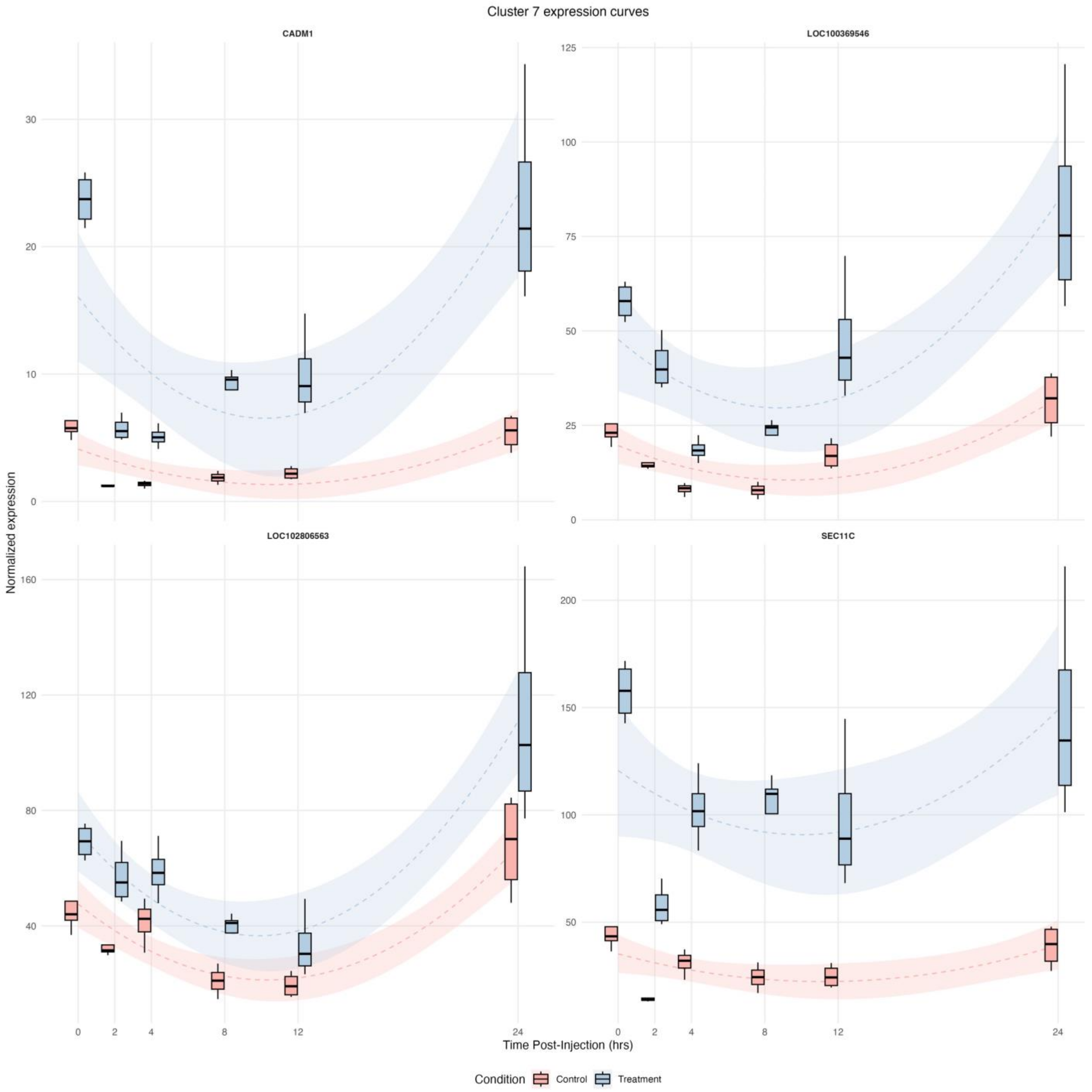

Figure S9. Distribution of quadratic regression curves fit to Cluster 7 pDEGs.

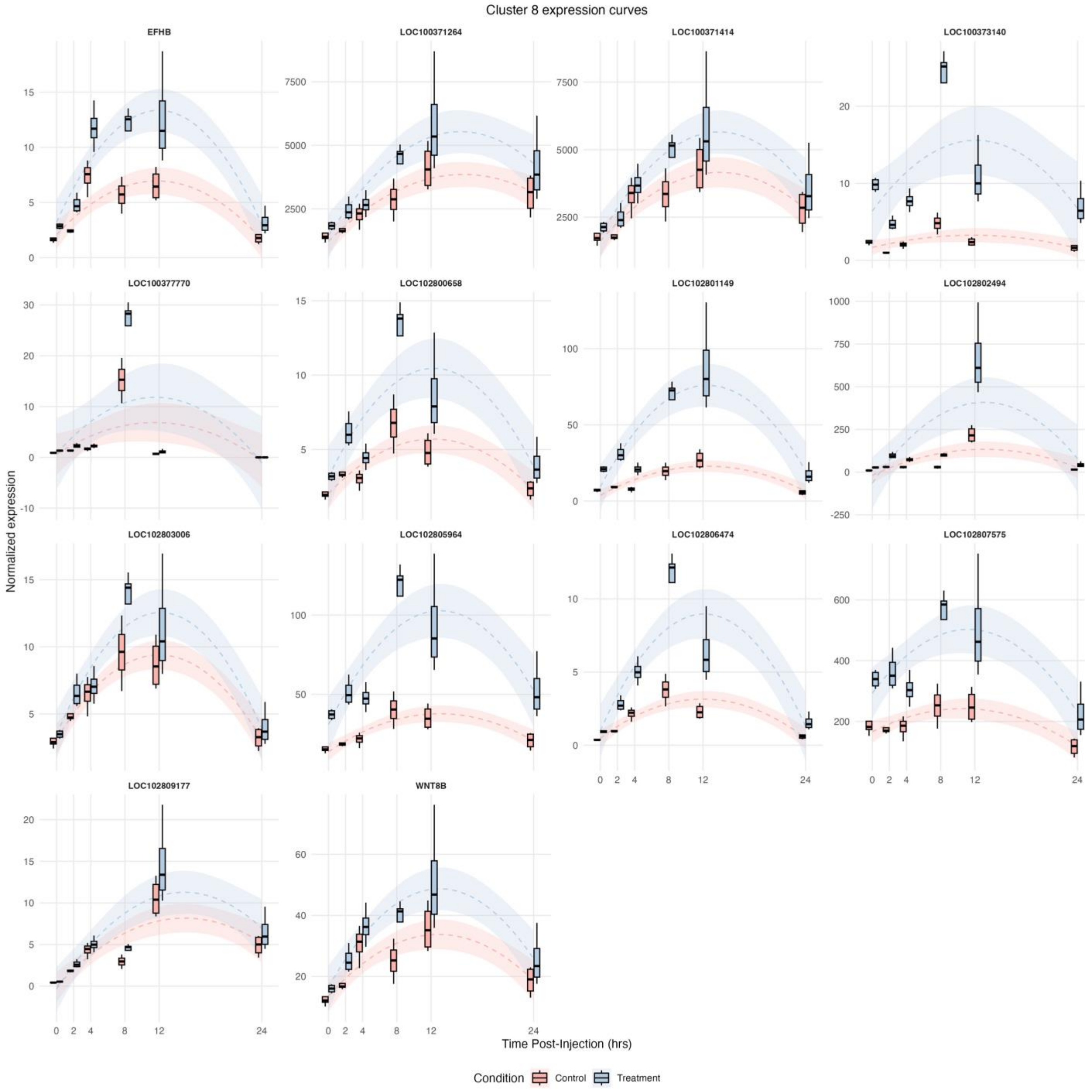

Figure S10. Distribution of quadratic regression curves fit to Cluster 8 pDEGs.

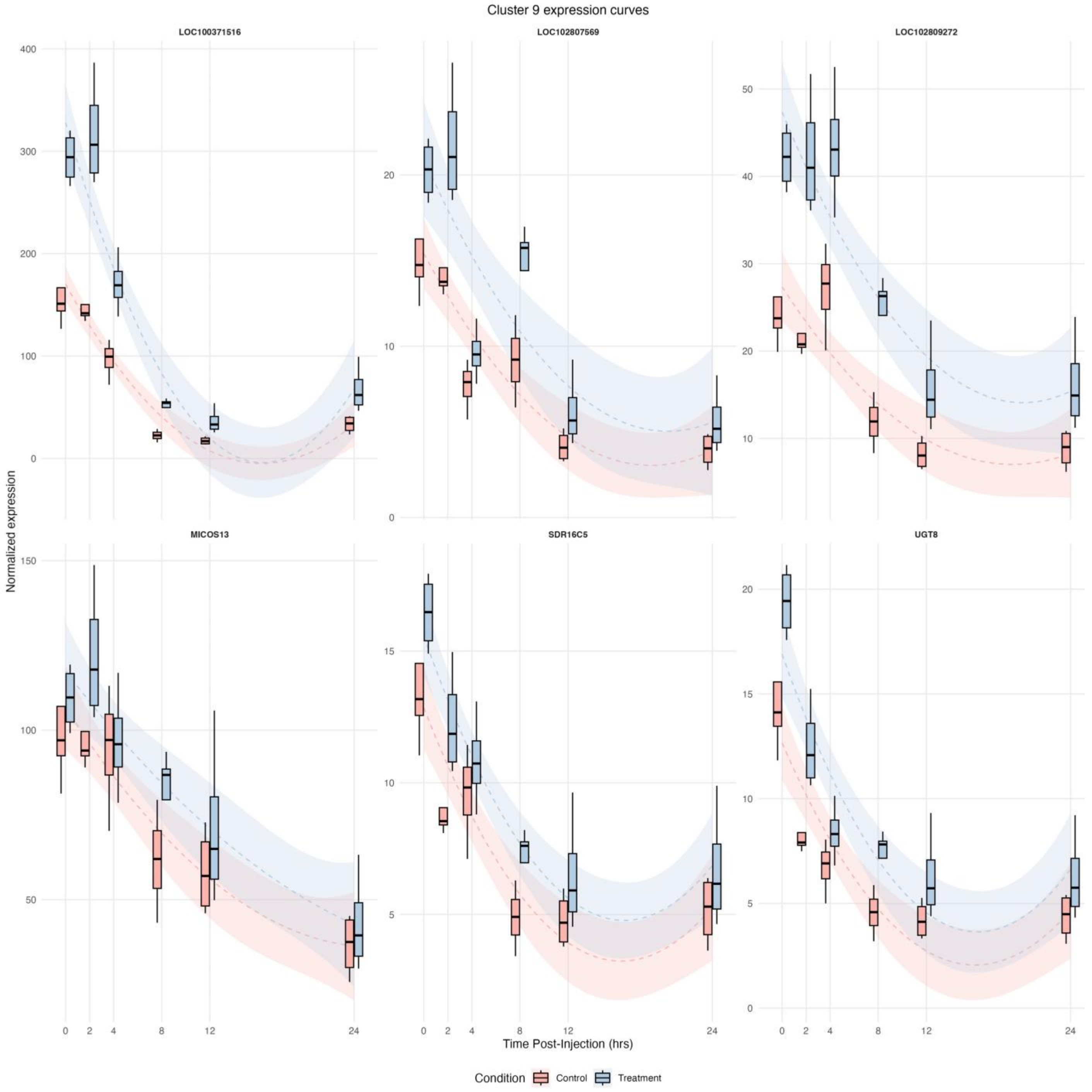

Figure S11. Distribution of quadratic regression curves fit to Cluster 9 pDEGs.

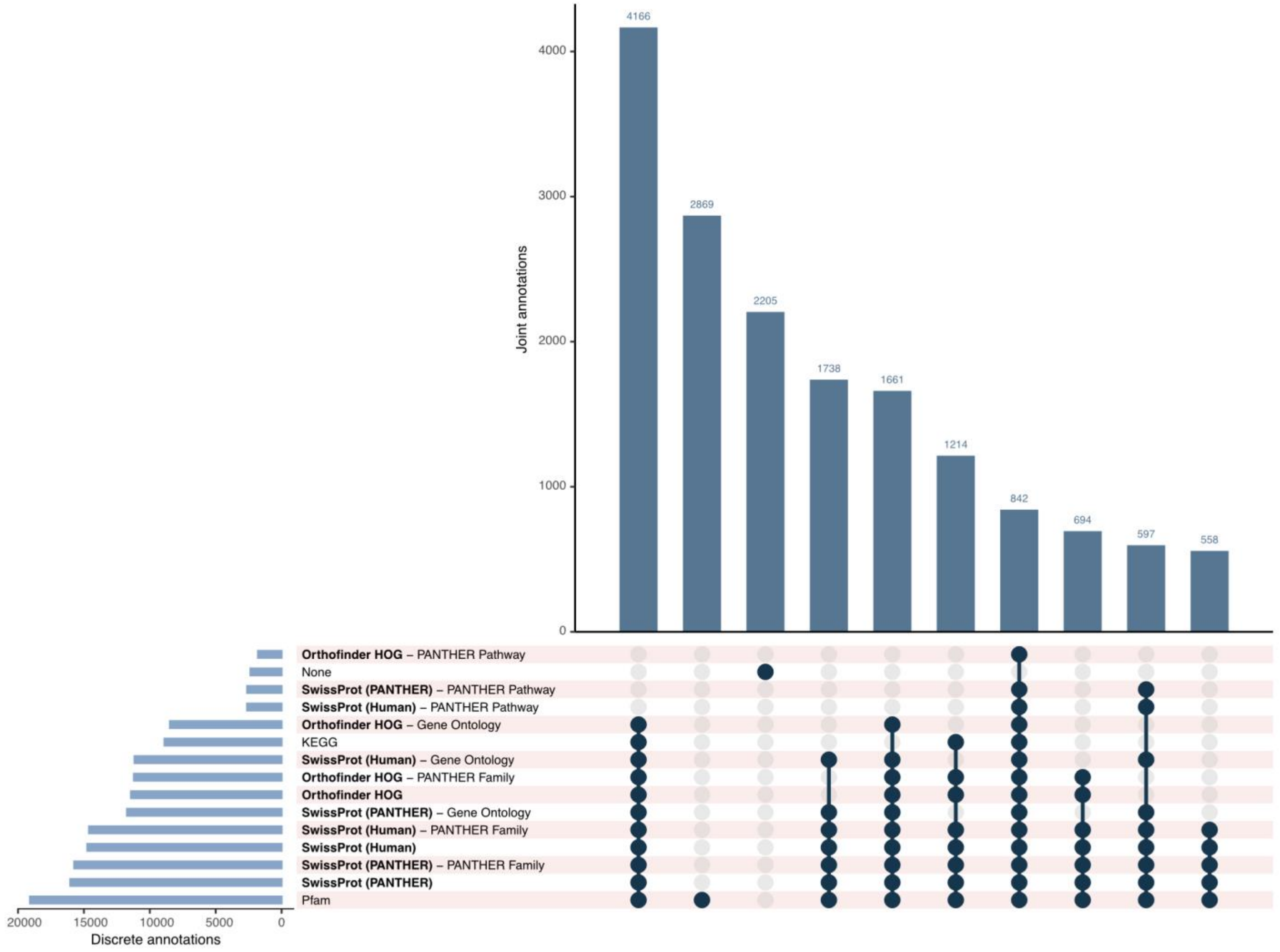

Figure S12. Annotation frequency of the *S. kowalevskii* genome. Left panel reflects the total number of genes annotated by each nonoverlapping annotation set, where bolded terms reflect the three parent annotation protocols. Top panel shows the top 10 sets of genes ranked by their joint annotation frequency (shown in the bottom right panel) across the *S. kowalevskii* genome. For example, the greatest set of genes were annotated by all methods except for PANTHER Pathway, followed by genes with Pfam annotations alone, no annotations, etc.

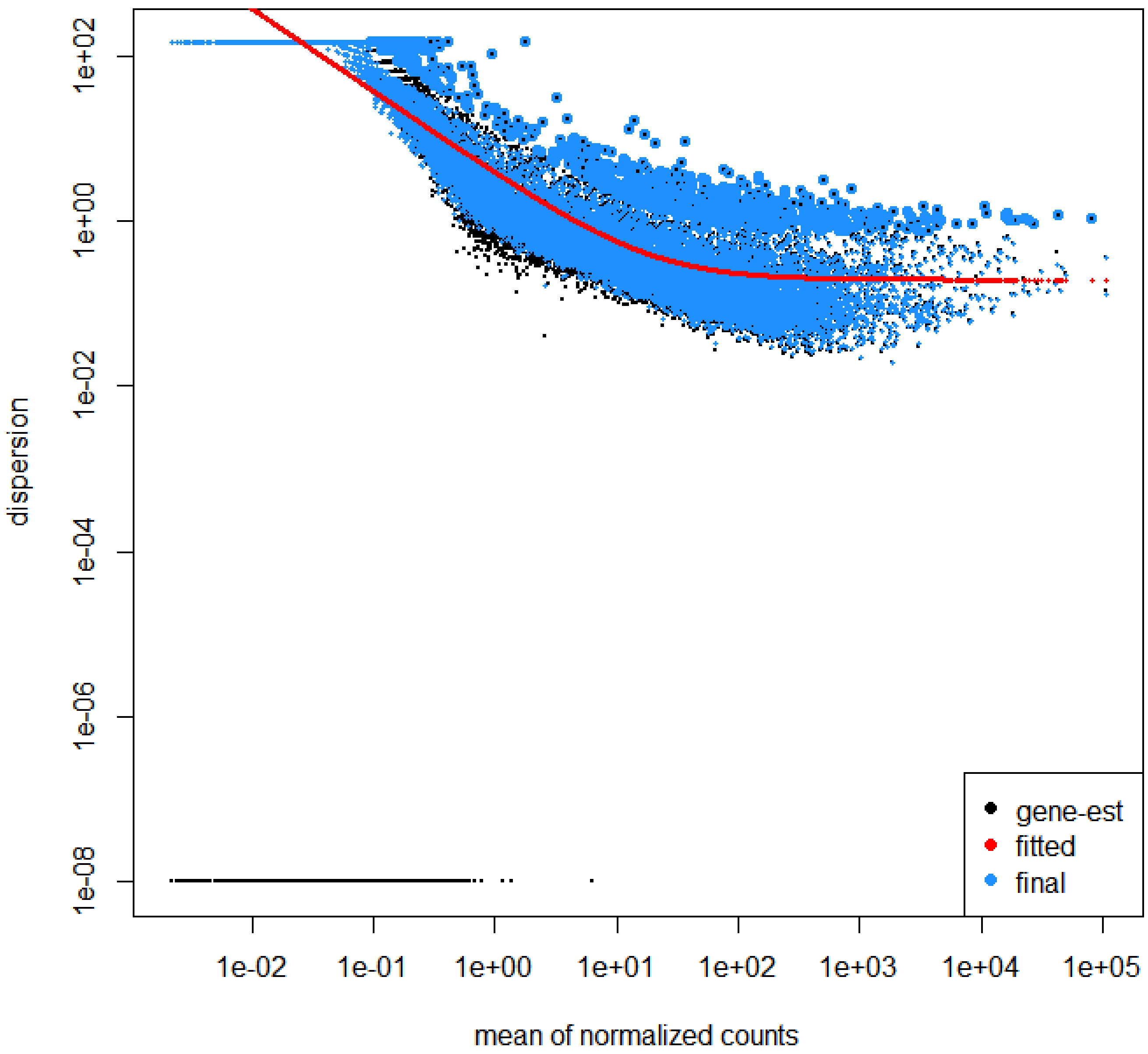

Figure S13. Dispersion plot for DESeq2 experiment showing per-gene dispersion estimates (black), fitted estimates of dispersion (red), and shrunken dispersion estimates for each gene (blue). Per-gene estimations encompassed by a blue circle indicate gene estimates that were not shrunken towards fitted dispersion curve.

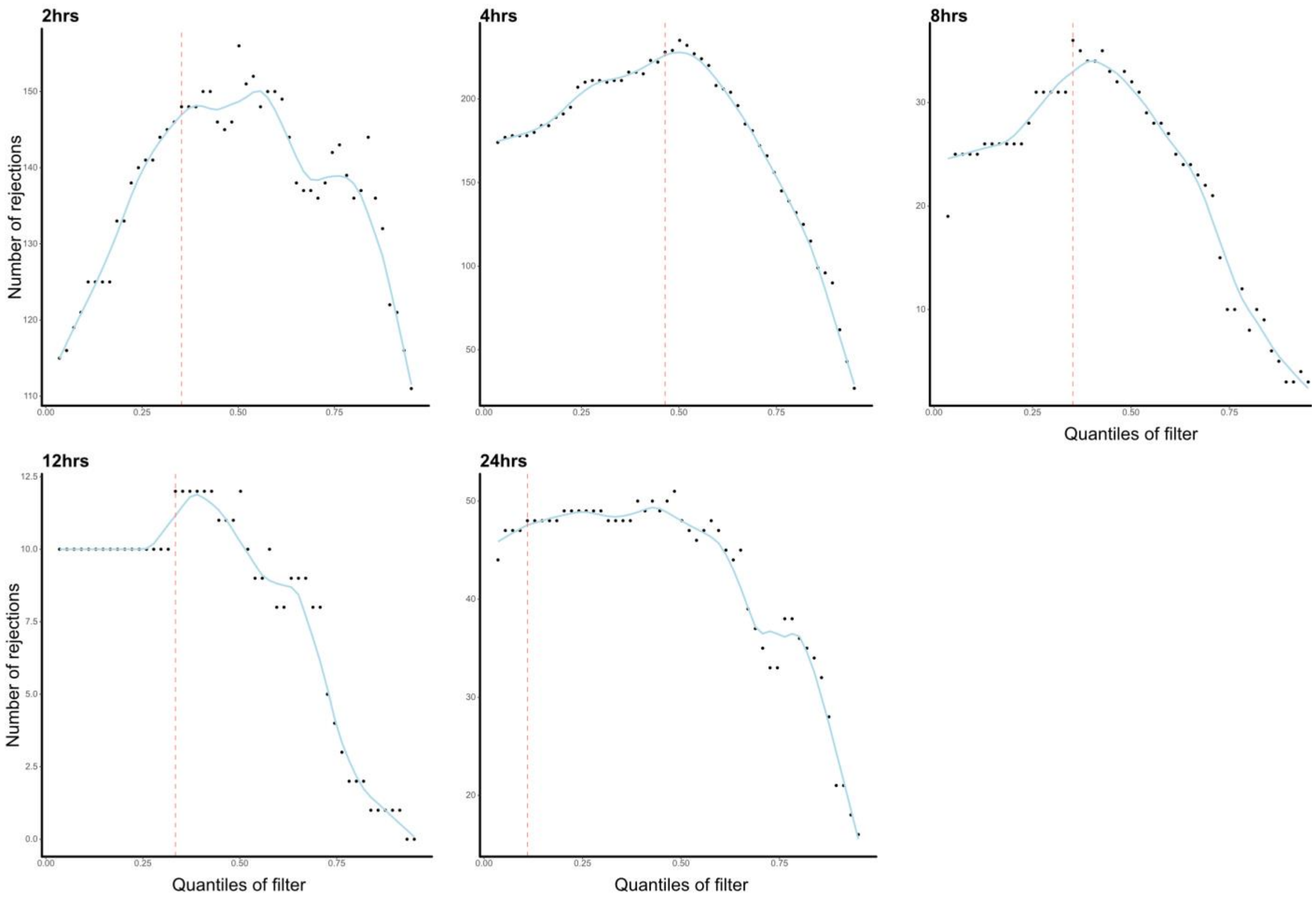

Figure S14. Independent filtering plots for each contrast (Treatment vs. control at each time point relative to 0hrs post-injection). Y-axis shows the number of p-value rejections above the specified false-discovery rate threshold ($\alpha = 0.05$); X-axis shows the ranked quantiles of mean normalized counts across all genes in the given contrast. Dotted line represents the lowest quantile of filter where the number of rejections is within one residual standard deviation of the peak of the curve fit to the number of rejections at the specified FDR. See **Table S2** for further details on independent filtering thresholds per contrast.

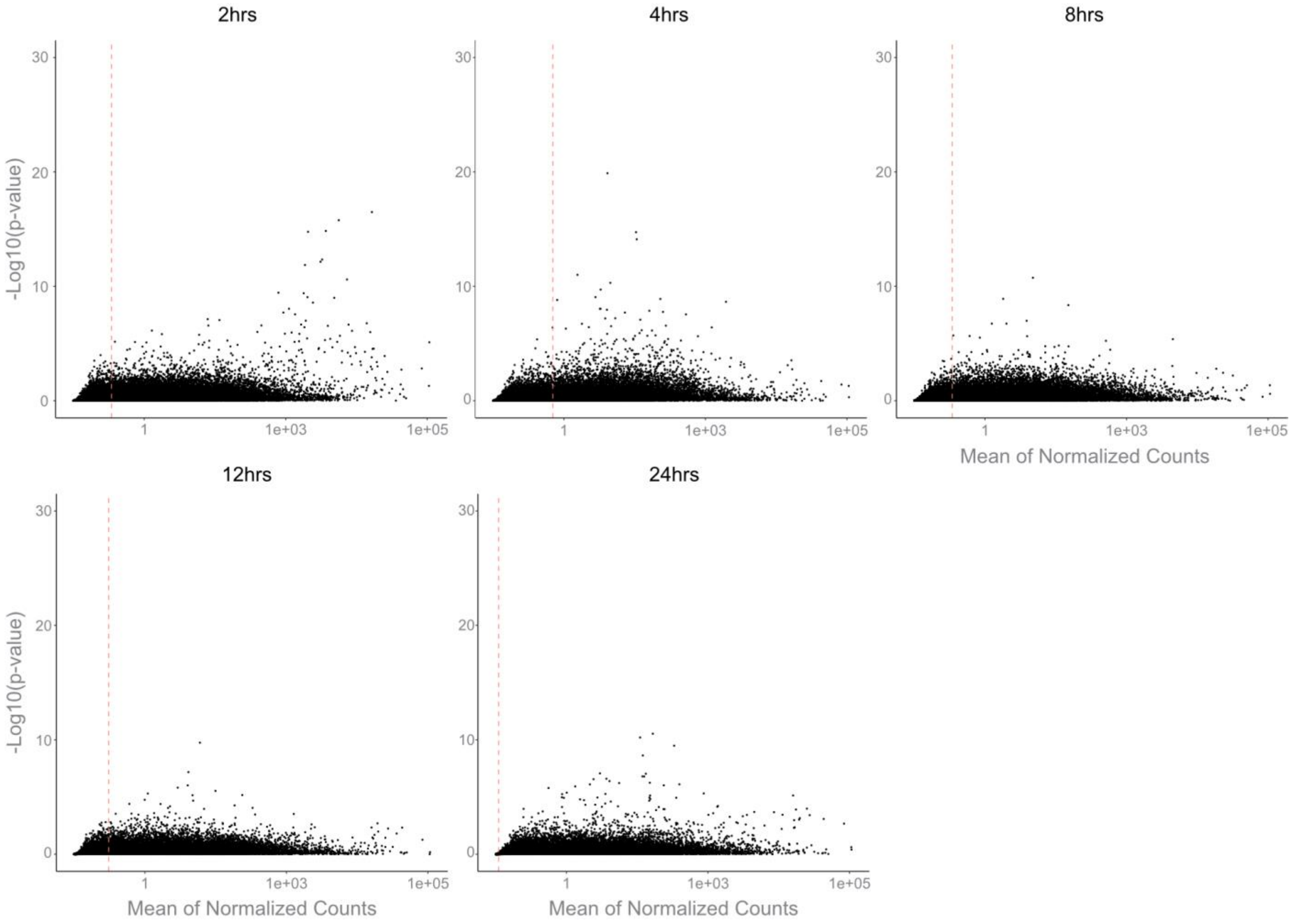

Figure S15. Plot showing the independent filtering cutoff for mean of normalized counts per-gene. Y-axis shows the -Log_{10}(p-value) for each gene in the contrast; X-axis is the log-scaled mean of normalized counts (+1 pseudocount) for each gene in the contrast. The dotted vertical line shows the independent filtering threshold corresponding to the same vertical line in **Figure S14**.

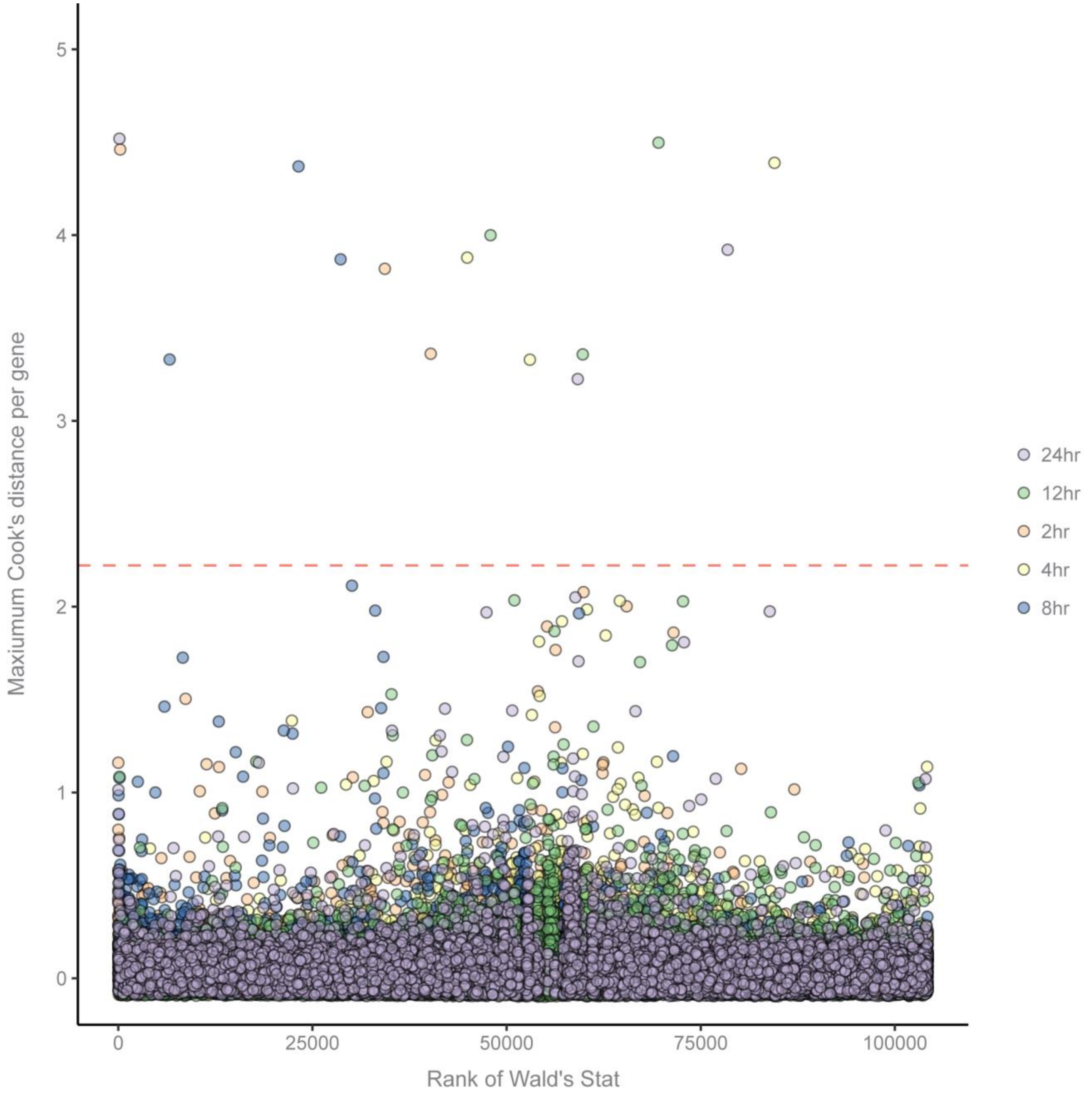

Figure S16. Plot showing the Cook’s distance cutoff for DESeq2 experiment. Cook’s distance cutoff (dashed line) is set by default as the 99% quantile of the F(p,m-p) distribution (where *p* is the number of parameters including the intercept, and *m* is the number of samples).

Table S1: Sample metadata, sequencing statistics, and mapping rates

	Metadata				Sequencing Reads			Mapping	
Sample	Rep*	Condition	Tissue	TPI	Raw	Cleaned	% Ret**	Unique	Multi
2C0C	2	Control	Collar	0	6513256	6167465	94.69%	4370017 (70.86%)	1640080 (26.59%)
2C0P	2	Control	Proboscis	0	6619666	6369347	96.22%	5952043 (93.45%)	262007 (4.11%)
2C0T	2	Control	Trunk	0	5337846	5206737	97.54%	4787075 (91.94%)	223568 (4.29%)
2T0C	2	Treatment	Collar	0	8259324	7954854	96.31%	6655264 (83.66%)	1021746 (12.84%)
2T0P	2	Treatment	Proboscis	0	5586058	5420449	97.04%	5108482 (94.24%)	191129 (3.53%)
2T0T	2	Treatment	Trunk	0	6619882	6329306	95.61%	5482857 (86.63%)	554899 (8.77%)
2C2C	2	Control	Collar	2	6408972	6081601	94.89%	5220510 (85.84%)	668559 (10.99%)
2C2P	2	Control	Proboscis	2	9861000	9147991	92.77%	7418897 (81.10%)	1504439 (16.45%)
2C2T	2	Control	Trunk	2	5850429	5586214	95.48%	4651997 (83.28%)	774876 (13.87%)
2T2C	2	Treatment	Collar	2	6154648	5869524	95.37%	3495613 (59.56%)	2213756 (37.72%)
2T2P	2	Treatment	Proboscis	2	8358600	8114891	97.08%	7467084 (92.02%)	479738 (5.91%)
2T2T	2	Treatment	Trunk	2	4743187	4593878	96.85%	4151569 (90.37%)	230594 (5.02%)
2C4C	2	Control	Collar	4	7776629	7514611	96.63%	6190966 (82.39%)	1127023 (15.00%)
2C4P	2	Control	Proboscis	4	7958525	7770086	97.63%	7077782 (91.09%)	530606 (6.83%)
2C4T	2	Control	Trunk	4	7856773	7721049	98.27%	7168792 (92.85%)	268139 (3.47%)
2T4C	2	Treatment	Collar	4	6575393	6293802	95.72%	5221827 (82.97%)	848670 (13.48%)
2T4P	2	Treatment	Proboscis	4	6344244	5945192	93.71%	5119037 (86.10%)	675931 (11.37%)
2T4T	2	Treatment	Trunk	4	7273025	7016244	96.47%	6259260 (89.21%)	418213 (5.96%)
2C8C	2	Control	Collar	8	5580484	5249739	94.07%	4064753 (77.43%)	1048030 (19.96%)
2C8P	2	Control	Proboscis	8	4651049	4249479	91.37%	3810908 (89.68%)	329442 (7.75%)
2C8T	2	Control	Trunk	8	6246604	5962549	95.45%	5118344 (85.84%)	582091 (9.76%)
2T8C	2	Treatment	Collar	8	5453671	5216084	95.64%	4537574 (86.99%)	538579 (10.33%)
2T8P	2	Treatment	Proboscis	8	6134518	5950218	97.00%	5625481 (94.54%)	180688 (3.04%)
2T8T	2	Treatment	Trunk	8	6433870	6136305	95.38%	5140862 (83.78%)	775107 (12.63%)
2C12C	2	Control	Collar	12	6913987	6548496	94.71%	3515271 (53.68%)	2841104 (43.39%)
2C12P	2	Control	Proboscis	12	9159487	8927213	97.46%	7496607 (83.97%)	1237093 (13.86%)
2C12T	2	Control	Trunk	12	5714963	5451287	95.39%	4810941 (88.25%)	437102 (8.02%)
2T12C	2	Treatment	Collar	12	6096237	5924673	97.19%	5458664 (92.13%)	291897 (4.93%)
2T12P	2	Treatment	Proboscis	12	5806881	5644299	97.20%	5185295 (91.87%)	323119 (5.72%)
2T12T	2	Treatment	Trunk	12	6958901	6636202	95.36%	5631918 (84.87%)	751803 (11.33%)
2C24C	2	Control	Collar	24	6444470	6019677	93.41%	4259508 (70.76%)	1602224 (26.62%)
2C24P	2	Control	Proboscis	24	6821810	6251237	91.64%	5510949 (88.16%)	584799 (9.35%)
2C24T	2	Control	Trunk	24	6825255	6563990	96.17%	5974563 (91.02%)	346034 (5.27%)
2T24C	2	Treatment	Collar	24	2354917	2236345	94.96%	1575813 (70.46%)	603378 (26.98%)
2T24P	2	Treatment	Proboscis	24	7679506	7079732	92.19%	6164599 (87.07%)	754846 (10.66%)
2T24T	2	Treatment	Trunk	24	6260388	5951618	95.07%	4918331 (82.64%)	818862 (13.76%)
3C0C	3	Control	Collar	0	7959684	7336677	92.17%	5361362 (73.08%)	1775294 (24.20%)
3C0P	3	Control	Proboscis	0	7907310	7326804	92.66%	6577277 (89.77%)	531793 (7.26%)
3C0T	3	Control	Trunk	0	7181077	6922738	96.40%	5093543 (73.58%)	1633244 (23.59%)
3T0C	3	Treatment	Collar	0	5690772	5408477	95.04%	3920033 (72.48%)	1321926 (24.44%)
3T0P	3	Treatment	Proboscis	0	7239449	6662025	92.02%	5722962 (85.90%)	781662 (11.73%)
3T0T	3	Treatment	Trunk	0	7130503	6907257	96.87%	5940785 (86.01%)	693213 (10.04%)
3C2C	3	Control	Collar	2	9274808	8952379	96.52%	4896091 (54.69%)	3810433 (42.56%)
3C2P	3	Control	Proboscis	2	6566317	6273093	95.53%	5781454 (92.16%)	347806 (5.54%)
3C2T	3	Control	Trunk	2	5837990	5668087	97.09%	5191878 (91.60%)	293999 (5.19%)
3T2C	3	Treatment	Collar	2	8028109	7521804	93.69%	5521721 (73.41%)	1781839 (23.69%)
3T2P	3	Treatment	Proboscis	2	7061698	6463452	91.53%	5687865 (88.00%)	612457 (9.48%)
3T2T	3	Treatment	Trunk	2	6926214	6734503	97.23%	6168230 (91.59%)	308365 (4.58%)
3C4C	3	Control	Collar	4	6390858	6143853	96.14%	3943033 (64.18%)	1973703 (32.12%)
3C4P	3	Control	Proboscis	4	5993608	5598079	93.40%	4923400 (87.95%)	516585 (9.23%)
3C4T	3	Control	Trunk	4	6564535	6347748	96.70%	5718327 (90.08%)	387765 (6.11%)
3T4C	3	Treatment	Collar	4	8556850	7888025	92.18%	6797949 (86.18%)	749843 (9.51%)
3T4P	3	Treatment	Proboscis	4	5968893	5770785	96.68%	5383439 (93.29%)	254049 (4.40%)
3T4T	3	Treatment	Trunk	4	5730831	5479068	95.61%	4812387 (87.83%)	399161 (7.29%)
3C8C	3	Control	Collar	8	5154655	4958781	96.20%	3341584 (67.39%)	1490293 (30.05%)

3C8P	3	Control	Proboscis	8	5807005	5342217	92.00%	4494142 (84.13%)	724794 (13.57%)
3C8T	3	Control	Trunk	8	8420164	8002595	95.04%	6343650 (79.27%)	1443964 (18.04%)
3T8C	3	Treatment	Collar	8	6632491	6297382	94.95%	4601590 (73.07%)	1455465 (23.11%)
3T8P	3	Treatment	Proboscis	8	10327251	9883725	95.71%	8784407 (88.88%)	895743 (9.06%)
3T8T	3	Treatment	Trunk	8	7457274	7228023	96.93%	6268530 (86.73%)	745845 (10.32%)
3C12C	3	Control	Collar	12	6148670	5932636	96.49%	4787838 (80.70%)	984910 (16.60%)
3C12P	3	Control	Proboscis	12	5768557	5606878	97.20%	5322743 (94.93%)	160805 (2.87%)
3C12T	3	Control	Trunk	12	5069516	4990290	98.44%	4645079 (93.08%)	156866 (3.14%)
3T12C	3	Treatment	Collar	12	7036687	6690400	95.08%	5370563 (80.27%)	1034916 (15.47%)
3T12P	3	Treatment	Proboscis	12	6351778	5999193	94.45%	5417998 (90.31%)	429705 (7.16%)
3T12T	3	Treatment	Trunk	12	6001892	5817068	96.92%	5332684 (91.67%)	260297 (4.47%)
3C24C	3	Control	Collar	24	6081225	5790525	95.22%	4158333 (71.81%)	1429633 (24.69%)
3C24P	3	Control	Proboscis	24	6628564	6432038	97.04%	6071478 (94.39%)	202682 (3.15%)
3C24T	3	Control	Trunk	24	7309285	7055457	96.53%	6333170 (89.76%)	486257 (6.89%)
3T24C	3	Treatment	Collar	24	5778315	5546276	95.98%	4210220 (75.91%)	1186209 (21.39%)
3T24P	3	Treatment	Proboscis	24	5308798	5157396	97.15%	4885209 (94.72%)	157901 (3.06%)
3T24T	3	Treatment	Trunk	24	4980744	4875991	97.90%	4533492 (92.98%)	144669 (2.97%)
D4C0C	4	Control	Collar	0	6868670	6477512	94.31%	4368309 (67.44%)	1862472 (28.75%)
D4C0P	4	Control	Proboscis	0	5620586	5233406	93.11%	4399361 (84.06%)	710442 (13.58%)
D4C0T	4	Control	Trunk	0	8706931	8337416	95.76%	6627191 (79.49%)	1242860 (14.91%)
D4T0C	4	Treatment	Collar	0	7988559	7544671	94.44%	5292331 (70.15%)	1979860 (26.24%)
D4T0P	4	Treatment	Proboscis	0	9063671	8407548	92.76%	6007608 (71.45%)	2201754 (26.19%)
D4T0T	4	Treatment	Trunk	0	5393736	5257154	97.47%	4768113 (90.70%)	289965 (5.52%)
D4C2C	4	Control	Collar	2	5713786	5331557	93.31%	3972782 (74.51%)	1187987 (22.28%)
D4C2P	4	Control	Proboscis	2	5290255	5135578	97.08%	4634383 (90.24%)	390185 (7.60%)
D4C2T	4	Control	Trunk	2	7247470	6992856	96.49%	6215177 (88.88%)	400884 (5.73%)
D4T2C	4	Treatment	Collar	2	8493454	8088639	95.23%	6581724 (81.37%)	1204269 (14.89%)
D4T2P	4	Treatment	Proboscis	2	8395474	8152251	97.10%	7012419 (86.02%)	984123 (12.07%)
D4T2T	4	Treatment	Trunk	2	4542746	4405781	96.98%	4050761 (91.94%)	170774 (3.88%)
D4C4C	4	Control	Collar	4	6006543	5707679	95.02%	4609339 (80.76%)	937726 (16.43%)
D4C4P	4	Control	Proboscis	4	8431385	8005446	94.95%	6851010 (85.58%)	970288 (12.12%)
D4C4T	4	Control	Trunk	4	4051456	3912466	96.57%	3538592 (90.44%)	230190 (5.88%)
D4T4C	4	Treatment	Collar	4	9133602	8626345	94.45%	5485546 (63.59%)	2772143 (32.14%)
D4T4P	4	Treatment	Proboscis	4	7456306	6869497	92.13%	5926302 (86.27%)	760706 (11.07%)
D4T4T	4	Treatment	Trunk	4	6438671	6171842	95.86%	5365641 (86.94%)	467423 (7.57%)
D4C8C	4	Control	Collar	8	8421559	8158439	96.88%	4931495 (60.45%)	2969969 (36.40%)
D4C8P	4	Control	Proboscis	8	7671829	7160221	93.33%	6372755 (89.00%)	567492 (7.93%)
D4C8T	4	Control	Trunk	8	6935579	6577132	94.83%	5226100 (79.46%)	894208 (13.60%)
D4T8C	4	Treatment	Collar	8	6460220	6274663	97.13%	2364013 (37.68%)	3798819 (60.54%)
D4T8P	4	Treatment	Proboscis	8	5362233	5195874	96.90%	4697446 (90.41%)	386600 (7.44%)
D4T8T	4	Treatment	Trunk	8	10294873	9945730	96.61%	8346686 (83.92%)	1198400 (12.05%)
D4C12C	4	Control	Collar	12	6538333	6121249	93.62%	5046539 (82.44%)	824174 (13.46%)
D4C12P	4	Control	Proboscis	12	6489481	5815248	89.61%	4782410 (82.24%)	857157 (14.74%)
D4C12T	4	Control	Trunk	12	7882213	7601848	96.44%	6752692 (88.83%)	476475 (6.27%)
D4T12C	4	Treatment	Collar	12	7615314	7291568	95.75%	5119583 (70.21%)	1947820 (26.71%)
D4T12P	4	Treatment	Proboscis	12	9172740	8495553	92.62%	7492987 (88.20%)	770470 (9.07%)
D4T12T	4	Treatment	Trunk	12	9285462	8862010	95.44%	7293881 (82.31%)	1250778 (14.11%)
D4C24C	4	Control	Collar	24	6441980	6161584	95.65%	5048131 (81.93%)	948399 (15.39%)
D4C24P	4	Control	Proboscis	24	6792552	6482955	95.44%	5964327 (92.00%)	382157 (5.89%)
D4C24T	4	Control	Trunk	24	7499029	7226370	96.36%	6258770 (86.61%)	682655 (9.45%)
D4T24C	4	Treatment	Collar	24	6888815	6738061	97.81%	6279929 (93.20%)	271243 (4.03%)
D4T24P	4	Treatment	Proboscis	24	6323963	6073715	96.04%	5698589 (93.82%)	208274 (3.43%)
D4T24T	4	Treatment	Trunk	24	9091657	8850005	97.34%	8127416 (91.84%)	373747 (4.22%)
D5C0C	5	Control	Collar	0	6741291	6428587	95.36%	4857687 (75.56%)	1382323 (21.50%)
D5C0P	5	Control	Proboscis	0	6936474	6738707	97.15%	6240966 (92.61%)	355332 (5.27%)
D5C0T	5	Control	Trunk	0	11939362	11603196	97.18%	10301594 (88.78%)	882578 (7.61%)
D5T0C	5	Treatment	Collar	0	9440731	8957886	94.89%	7005343 (78.20%)	1703204 (19.01%)
D5T0P	5	Treatment	Proboscis	0	6672742	6515341	97.64%	6131493 (94.11%)	255979 (3.93%)

D5T0T	5	Treatment	Trunk	0	5619606	5393752	95.98%	4907263 (90.98%)	263109 (4.88%)
D5C2C	5	Control	Collar	2	8345630	8205253	98.32%	2598378 (31.67%)	5456905 (66.51%)
D5C2P	5	Control	Proboscis	2	6717209	6541916	97.39%	6085430 (93.02%)	315147 (4.82%)
D5C2T	5	Control	Trunk	2	8313949	7961581	95.76%	6636493 (83.36%)	914837 (11.49%)
D5T2C	5	Treatment	Collar	2	9281689	8885460	95.73%	7740369 (87.11%)	930236 (10.47%)
D5T2P	5	Treatment	Proboscis	2	6935958	6741867	97.20%	6038532 (89.57%)	580623 (8.61%)
D5T2T	5	Treatment	Trunk	2	6629357	6366757	96.04%	5719072 (89.83%)	390444 (6.13%)
D5C4C	5	Control	Collar	4	8968741	8579678	95.66%	6736223 (78.51%)	1619027 (18.87%)
D5C4P	5	Control	Proboscis	4	8959151	8616720	96.18%	8042917 (93.34%)	396014 (4.60%)
D5C4T	5	Control	Trunk	4	9526634	9217766	96.76%	7887907 (85.57%)	1005780 (10.91%)
D5T4C	5	Treatment	Collar	4	9546943	8995323	94.22%	7015907 (78.00%)	1693093 (18.82%)
D5T4P	5	Treatment	Proboscis	4	7152826	6493425	90.78%	5442802 (83.82%)	868433 (13.37%)
D5T4T	5	Treatment	Trunk	4	9116102	8760066	96.09%	6859052 (78.30%)	1625778 (18.56%)
D5C8C	5	Control	Collar	8	9917822	9350048	94.28%	7684994 (82.19%)	1367868 (14.63%)
D5C8P	5	Control	Proboscis	8	7299196	6797345	93.12%	5616280 (82.62%)	994830 (14.64%)
D5C8T	5	Control	Trunk	8	6884499	6666754	96.84%	5948638 (89.23%)	455711 (6.84%)
D5T8C	5	Treatment	Collar	8	8450871	8153545	96.48%	5675398 (69.61%)	2240049 (27.47%)
D5T8P	5	Treatment	Proboscis	8	8510535	8141322	95.66%	7553406 (92.78%)	397634 (4.88%)
D5T8T	5	Treatment	Trunk	8	8964101	8532158	95.18%	7295918 (85.51%)	823004 (9.65%)
D5C12C	5	Control	Collar	12	7972181	7772655	97.50%	6954625 (89.48%)	613612 (7.89%)
D5C12P	5	Control	Proboscis	12	10554566	10195779	96.60%	9216872 (90.40%)	751906 (7.37%)
D5C12T	5	Control	Trunk	12	8686478	8157965	93.92%	6530827 (80.05%)	1301587 (15.95%)
D5T12C	5	Treatment	Collar	12	7033179	6669278	94.83%	5886725 (88.27%)	552411 (8.28%)
D5T12P	5	Treatment	Proboscis	12	4848823	4701432	96.96%	4219772 (89.76%)	373781 (7.95%)
D5T12T	5	Treatment	Trunk	12	8402106	8110750	96.53%	6858394 (84.56%)	912999 (11.26%)
D5C24C	5	Control	Collar	24	6943749	6701190	96.51%	4105884 (61.27%)	2360975 (35.23%)
D5C24P	5	Control	Proboscis	24	6734130	6058386	89.97%	5014630 (82.77%)	881669 (14.55%)
D5C24T	5	Control	Trunk	24	7812330	7412088	94.88%	5146666 (69.44%)	1579097 (21.30%)
D5T24C	5	Treatment	Collar	24	6701966	6352876	94.79%	5469281 (86.09%)	690669 (10.87%)
D5T24P	5	Treatment	Proboscis	24	7322777	6894277	94.15%	6199253 (89.92%)	500174 (7.25%)
D5T24T	5	Treatment	Trunk	24	6656478	6321918	94.97%	4799281 (75.91%)	1245145 (19.70%)

*Replicate 1 was omitted as a test trial as it was not included in any analysis reported in this study
**Percentage of reads which retained after *fastp* quality filter

Table S2: Independent filtering metrics per contrast

Timepoint	Quantile Threshold	Mean Count Threshold	# Genes Removed
2hrs	35.27%	2.44	6852
4hrs	46.47%	5.96	9272
8hrs	35.27%	2.44	6852
12hrs	33.41%	2.07	6448
24hrs	11.01%	0.10	1614
